# Theory of Cation Solvation in the Helmholtz Layer of Li-Ion Battery Electrolytes

**DOI:** 10.1021/acsaem.5c00883

**Published:** 2025-06-02

**Authors:** Zachary A. H. Goodwin, Daniel M. Markiewitz, Qisheng Wu, Yue Qi, Martin Z. Bazant

**Affiliations:** † Department of Materials, University of Oxford, Parks Road, Oxford OX1 3PH, United Kingdom; ‡ John A. Paulson School of Engineering and Applied Sciences, Harvard University, Cambridge, Massachusetts 02138, United States; § Department of Chemical Engineering, Massachusetts Institute of Technology, Cambridge, Massachusetts 02139, United States; ∥ School of Engineering, Brown University, Providence, Rhode Island 02912, United States; ⊥ Department of Mathematics, Massachusetts Institute of Technology, Cambridge, Massachusetts 02139, United States

**Keywords:** electric double layer, battery electrolytes, solvation, ionic association, Helmholtz

## Abstract

The solvation environments of Li^+^ in conventional nonaqueous battery electrolytes, such as LiPF_6_ in mixtures of ethylene carbaronate (EC) and ethyl methyl carbonate (EMC), are often used to rationalize transport properties and solid electrolyte interphase (SEI) formation. Solvation environments in the compact electrical double layer (EDL) next to the electrode, also known as the Helmholtz layer, determine (partially) what species can react to form the SEI, with bulk solvation environments often being used as a proxy. Here, we develop and test a theory of cation solvation in the Helmholtz layer of nonaqueous Li-ion battery electrolytes. First, we validate the theory against bulk and diffuse EDL atomistic molecular dynamics (MD) simulations of LiPF_6_ EC/EMC mixtures as a function of surface charge, where we find the theory can qualitatively capture the solvation environments. Next, we turn to the Helmholtz layer, where we find the main effect of the solvation structures next to the electrode is an apparent reduction in the number of binding sites between Li^+^ and the solvents, again where we find reasonable agreement with our developed theory. Finally, by solving a simplified version of the theory, we find that the probability of Li^+^ binding to each solvent remains equal to the bulk probability, suggesting that the bulk solvation environments are a reasonable place to start when understanding battery electrolytes. Our developed formalism can be parametrized from bulk MD simulations and used to predict the solvation environments in the Helmholtz layer through reducing the number of available coordination sites, which can be used to determine what could react and form the SEI.

## Graphical abstract

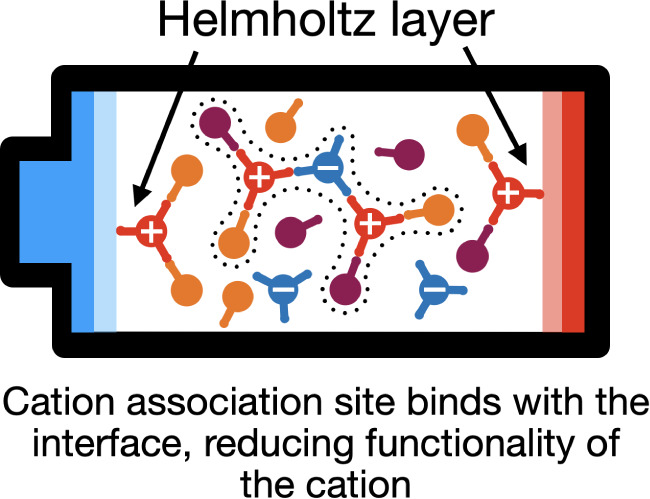

## Introduction

Lithium-ion batteries are set to play a central role in our efforts to decarbonize transportation and the storage of locally produced renewable energy.^1–4^ One of the central components of a Li-ion battery is the liquid electrolyte that transports the Li^+^ between the cathode and anode to store/release energy.^2,5^ The electrolytes that are used typically contain fluorinated anions, such as PF_6_^–^, and carbonate-based solvents, such as ethylene carbonate (EC) and ethyl methyl carbonate (EMC), with a salt concentration of ∼1 M.^6–9^ The carbonate solvents strongly interact with and solvate the Li^+^ ions through the carbonyl functional group, which regulates ionic aggregates at this relatively high salt concentration, and therefore, ensures good transport properties.^10–14^ One of the key observations in the field of battery electrolytes is the link between the solvation environments of active cations and the physiochemical properties, such as conductivity, transference numbers and formation of the solid electrolyte interphase (SEI), which is correlated with the long-term cycling ability of batteries.^10,13–18^ As bulk solvation environments are readily accessible from experiments^11,19–24^ and simulations^10–15,20,25–34^ these are often used as a starting point to understand battery electrolytes.^35^

However, what reacts at the electrode and forms the SEI is linked to the composition of the electrolyte at the charged interface, which is typically not the same as the bulk composition.^11,14,36,37^ More generally, without reactions, this is known as the electrical double layer (EDL) of the electrolyte^38–40^ with the electrolyte directly in contact with the electrode often being referred to as the (inner/outer) Helmholtz layer (or Stern layer), and the diffuse EDL is the distribution of the electrolyte which screens the remaining charge of the electrode, as depicted in Figure 1. In the context of conventional nonaqueous battery electrolytes, a large body of literature exists on simulating the EDL with atomistic methods, such as classical molecular dynamics (MD) and *ab initio* MD, where changes in composition of the electrolyte and solvation environments have been rationalized and used to interpret SEI formation.^11,14,36^ This area is further burgeoning with machine learning interatomic potentials^41–45^ and reaction networks,^46,47^ which have given great insight into SEI formation already.

**Figure 1:**
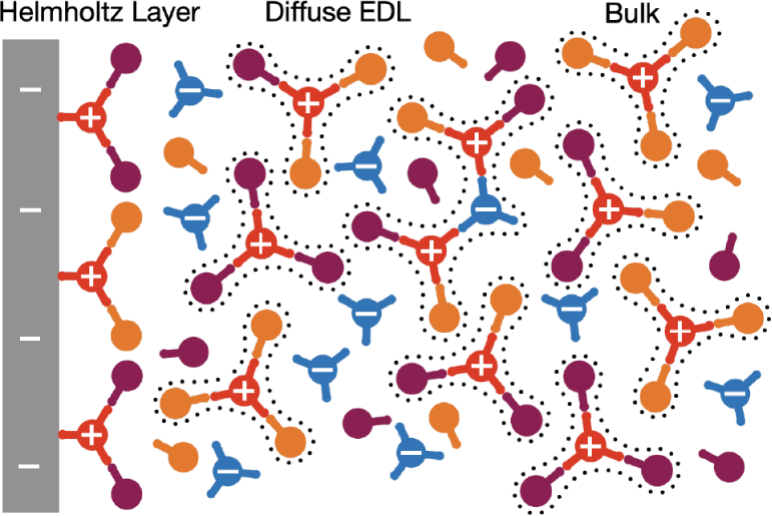
Schematic of conventional nonaqueous battery electrolytes in the bulk, where cation-solvation environments are depicted, in the diffuse electrical double layer (EDL), where larger aggregates are shown, and finally close to the interface there is the Helmholtz layer, where we have shown the cations interacting directly with the surface. One of the main parameters of the developed theory is the functionality of each species, i.e., the maximum number of associations it can form with other species. These functionalities are indicated as the sticks coming out of the circles for each species. For cations (denoted by at +) we have shown a functionality of 3, for anions (denoted with a −) we have again used 3, and the two solvents (distinguished by different colors) have a functionality of 1. We assume cation-solvent and cation–anion interactions are the only ones which dominate. At the Helmholtz layer, we find the interface blocks/binds to at least one of the cation association sites.

The EDL of electrolytes has a long history of being studied with relatively simple continuum thermodynamic theories.^38,39^ There has been success applying Bikerman-type models to predict capacitance responses and kinetics of charging electrodes, for example.^38,39^ Beyond simple local density approximations, there been great success modeling the structure of ionic liquids at the interface, where overscreening can be captured with a Bazant–Storey–Kornyshev^48^ theory reasonably well, or more accurately with weighted density approaches.^49^ In the context of battery electrolytes, however, this area appears to be less well developed, as the important solvation structures are not explicitly described with such simple EDL theories.^32^ Specifically, the solvation effects are often only included through assuming some effective radius of the ions, which is completely rigid, and cannot change composition/size. Therefore, a different approach to these classical theories is required, where specific interactions between ions and solvent can be accounted for reversibly.

Recently, McEldrew and Goodwin et al.^32,50–55^ have applied the reversible polymerization theories of Flory, Stockmayer and Tanaka to concentrated electrolytes, where ionic aggregation and solvation have been rationalized with a simple, analytical theory in the bulk and in the EDL. Moreover, Markiewitz et al. have extended this theory to the EDL of several realistic electrolytes.^56–58^ However, Markiewitz et al.^56^ found that the largest deviation between their theory and MD simulation occurred right at the interface, i.e., in the Helmholtz layer. Therefore, further development of this theory for the Helmholtz layer is needed^54^ and Li-ion battery electrolytes are an interesting system to start with because there are significant implications and applications for SEI formation.

In this paper, we develop and test a simple theory for the composition of the Helmholtz (or compact) double layer in conventional, nonaqueous Li-ion battery electrolyte mixtures. This theory is motivated from observations made from further analyzing the MD simulations performed by Wu et al. in ref. 14, where we find the main effect on the solvation structure in the Helmholtz layer is to reduce the number of available binding sites of Li^+^, i.e., the surface blocks/binds to one or more of the available solvation sites of Li^+^. First, we validate the bulk and diffuse EDL solvation environments against our theory, where we find good agreement, before moving onto the Helmholtz layer. By solving a simplified version of the theory in the Helmholtz layer, we find that the probability of Li^+^ binding to the solvents remains constant and equal to the bulk value, at least in the assumptions of this simplified theory. Therefore, we find some theoretical foundation as to why studying bulk solvation environments is a reasonable starting point for Li-ion battery electrolytes.

## Methods

Here we further analyze the molecular dynamics simulations of several conventional battery electrolytes investigated by Wu et al.^14^ Therefore, we refer the readers to ref. 14 for the details of the MD simulations. Here the EDL simulations are analyzed in 3 sections: bulk, diffuse EDL and Helmholtz layer. The bulk region as defined as the middle 20 Å region (the distance between the two electrodes was set to around 100 Å), the diffuse EDL is defined as from 5 Å from the interface to 10 Å from the interface, and the Helmholtz layer is defined from species at the interface to 5 Å (since this is the first layer of electrolyte in contact with the interface), as depicted in Figure 1. These choices for the demarcation between Helmholtz layer and diffuse EDL were made from observations of the EDL structure, where the Helmholtz layer was chosen as the layer directly in contact with the electrode, and the diffuse part is the remaining EDL. Note these values could be different for other electrolytes and surface charges, as the sizes of species will determine their layer sizes.

Within these regions, we extract the numbers of each species, and define an association between Li^+^ and F in PF_6_ from a real-space cutoff of 2.8 Å, and Li^+^ and O (carbonyl) in different solvents from a real-space cutoff of 2.8 Å.^14^ These real-space cutoffs were determined from inspecting the respective *g*(*r*) calculations. For Li^+^–O (carbonyl), the first pronounced peak is at just over 2 Å, and there is clear minimum at 2.8 Å which makes using this value for a real-space cutoff robust, as small changes in its value does not yield significant changes in coordination structure. For the Li^+^–F *g*(*r*), there is a small peak just above 2 Å and a slight minimum around 3 Å, which makes the definition of a real-space cutoff more arbitrary for these ionic associations, but we choose 2.8 Å as the value here. The structure in these *g*(*r*)’s clearly indicates that the coordination environments are important and well-defined for solvation, with the ionic associations being secondary and less well-defined. These definitions of associations are then used to compute coordination environments and the number of each aggregate in these regions. More details of this theoretical framework can be found in refs. 32, 52 ,^58^. Computing these associations allows us to investigate the essential components that a theory must have to be able to describe solvation in these different regions.

In this paper, we compare the MD determined cluster/solvent distributions against our theory in these different regions. As the bulk and diffuse EDL theory have been presented elsewhere, we refer the readers to refs. 32,50–58 and the Supporting Information for further details, and we will only provide an overview of the necessary equations and assumptions of the theory here. The central quantity that we are computing is the cluster/solvent distribution, as seen by

1 $$c_{lmsq}=\frac{W_{lmsq}}{\lambda_{-}}{\left(\psi_{l}\lambda_{-}\right)}^{l}{\left(\psi_{m}\lambda_{-}\right)}^{m}{\left(\psi_{s}\lambda_{x}\right)}^{s}{\left(\psi_{q}\lambda_{y}\right)}^{q}$$

Here *c*_*lmsq*_ is the dimensionless concentration of a cluster of rank *lmsq*, which means there are *l* cations, *m* anions, *s* solvent molecules of the first type (*x*) and *q* solvent molecules of the second type (*y*) bound together in an aggregate. The other terms in this equation will be explained in more detail shortly, but briefly *W*_*lmsq*_ is related to the multiplicity, λ_*j*_’s are the association constants and ψ_*j*_’s are related to the dimensionless concentration of free binding sites.

The dimensionless concentration is determined from *c*_*lmsq*_ = *N*_*lmsq*_/Ω, where *N*_*lmsq*_ is the number of clusters of that rank and

2 $$\Omega =\underset{lmsq}{\sum }\left(l+\xi_{-}m+\xi_{x}s+\xi_{y}q\right)N_{lmsq}$$

is the number of lattice sites occupied by the aggregates, where a single lattice site is set to the volume of the Li^+^ cation (*v*_+_), with ξ_*i*_ = *v_i_*/*v*_+_ being the volume ratio of each species to the Li^+^ cation, and *i* = *+,–,x,y*. Dividing through by the total number of lattice sites gives

3 $$1=\underset{lmsq}{\sum }\left(l+\xi_{-}m+\xi_{x}s+\xi_{y}q\right)c_{lmsq}=\underset{lmsq}{\sum }\phi_{lmsq}$$

which is a statement of incompressibility in the theory, where ϕ_*lmsq*_ is the volume fraction of a cluster of rank *lmsq*. It is also useful to know that the volume fraction of each species is determined through

4 $$\phi_{i}=\underset{lmsq}{\sum }\xi_{i}jc_{lmsq}$$

where *j* = *l,m,s,q*, with the number of each species being determined from

5 $$N_{i}=\underset{lmsq}{\sum }jN_{lmsq}$$

In our theory, we assume that Cayley-tree like aggregates form, which means no loops can exist, i.e., all of the aggregates are branched, as seen in Figure 1. This is to ensure an analytically tractable theory, as the free energy of the associations can be uniquely determined from the number of species in the aggregates.^32,50^ To form these Cayley-tree aggregates, we have to assume some maximum number of associations that the species can form, which we refer to as the functionality of the species, *f*_*i*_. For cations and anions it is kept general (*f*_+_ and *f*_–_, respectively), but for solvent we assume that only 1 association with the cation may form (no anion-solvent interactions). This is particularly reasonable in Li-salt electrolytes, as the cation is small and binds with other species strongly, while the anion and solvent interactions are weaker.^30,32^ These assumptions have been verified for conventional battery electrolytes and other electrolytes.^14,30,32^ For further information on these assumptions, we refer the reader to refs. 32,50–53 and the Discussion section.

In eq 1, the next term is given by

6 $$W_{lmsq}=\frac{\left(f_{+}l-l\right)!\left(f_{-}m-m\right)!}{l!m!s!q!\left(f_{+}l-l-m-s-q+1\right)!\left(f_{-}m-m-l+1\right)!}$$

which is related to the number of ways of arranging an aggregate of rank *lmsq* (see Supporting Information for more details). The λ_*i*_’s in eq 1 are the association constants, as seen by

7 $$\lambda_{i}=e^{-\beta \Delta f_{+i}}$$

where Δ*f*_+*i*_ is the free energy of formation of an association between cations and *i*, with the reference state being the free species in solution^50^ and β is the inverse thermal energy. Finally, ψ_*i*_ = *f*_*i*_ϕ_i_α_*i*_/ξ_*i*_ is the number of free association sites per lattice site for that species, where α_*i*_ is the fraction of that species that is free.

The problem we now face is that we wanted to determine α_*i*_ from our theory, not have it be an input for the theory. To overcome this, we follow Tanaka and introduce association probabilities and their corresponding mass-action laws.^50^ Therefore, we introduce $\alpha_{i}={\left(1-\underset{i^{\prime }}{\sum }p_{ii^{\prime }}\right)}^{f_{i}}$ , where $p_{ii^{\prime }}$ is the probability that *i* is associated with *i*′. These probabilities are related through the conservation of associations

8 $$\psi_{+}p_{+i}=\psi_{i}p_{i+}=\Gamma_{i}$$

where Γ_*i*_ is the number of +*i* associations per lattice site, ψ_*i*_ = *f_i_* ϕ_*i*_/ξ_*i*_ is analogous to that defined earlier but with the volume fraction of free species replaced with the total volume fraction of that species, and the mass action laws

9 $$\lambda_{i}\Gamma_{i}=\frac{p_{i+}p_{+i}}{\left(1-\underset{i^{\prime }}{\sum }p_{+i^{\prime }}\right)\left(1-p_{i+}\right)}$$

From solving this system of equations, the cluster distribution can be computed from the theory. All that is needed is the number of each species, *N*_*i*_, the assumed functionalities for each species, *f*_*i*_, the volume ratios, ξ_*i*_ (these are known from electrolyte composition), and to determine the association constant’s, λ_*i*_. Fortunately, the λ_*i*_’s can be determined from the MD simulations.^51–53^ First, the ensemble average coordination numbers of species associating to the cation are determined, which can then be divided by the cation functionality to find the association probabilities. The conservation of associations and mass action laws are then used to find the association constants.

Note that in ref. 54, it was shown that the same form of the cluster distribution should hold in the diffuse EDL, but where the quantities are replaced by their EDL counterparts, which is indicated with a bar. This theory was extended by Markiewitz et al.^56–58^ to describe WiSE, and in general more realistic electrolytes. In the Supporting Information we again show the Helmholtz layer should also follow this cluster distribution, but where the volume fractions, association probabilities and association constants can be different from the bulk/diffuse EDL. Here we compare the theory and MD simulations through computing the λ_*i*_ using the *N*_*i*_’s in the different regions for the most direct comparison. This means good agreement should be expected, but this allows us to verify the underlying assumptions of the theory and discover any new assumptions required for the Helmholtz layer.

From this full version of the theory, several assumptions can be made to investigate a simpler set of equations. First, the Li–PF_6_ associations are often weaker than the Li–solvent interactions, and in the Helmholtz layer at large negative surfaces charges in the diffuse EDL there are not many anions in the EDL, which means that, as a first approximation, the solvation properties can be focused on without the inclusion of cation–anion aggregation effects. This can be introduced though setting λ_–_ = 0 or from removing the association probabilities (*p*_–+_ and *p*_+–_) from the equations.^32^ Second, the sticky-cation assumption can be employed, where 1 = *p*_+*x*_ + *p*_+*y*_, i.e., the solvation shell of Li^+^ is full. The reader is referred to ref. 32 for more details. When these approximations are employed later on, the details will be provided.

## Results and Discussion

Here we show results for the 1 M LiPF_6_ in EC–EMC 3:7 volume ratio electrolyte. In the Supporting Information we show equivalent results for the other electrolytes investigated in ref. 14. Moreover, in the main text, we only focus on the solvation properties of Li^+^, not focusing on any ionic aggregation effects. In the Supporting Information, we show additional results for the comparison of the ionic aggregation in the bulk and diffuse EDL, as well as the other electrolytes investigated in ref. 14.

### Bulk

In Figure 2a we show *c*_10*sq*_, the concentration of the different solvation environments of Li^+^, as a function of the number of solvating EC (*s*) and EMC (*q*) from the MD simulations, with the average number of solvents coordinated to Li^+^ being 4.27. As seen, the most probable solvation structure is with 2EC + 2EMC [*s* = 2 and *q* = 2, as seen in (i) of Figure 2] solvating Li^+^. The next most probable solvation environments are found to be 3EC + 1 – 2EMC [i.e., *s* = 3 and *q* = 1–2, as seen in (ii) and (iii) of Figure 2]. We also find that there is some probability of solvation environments containing 4EC + EMC (from hereon out, we will interchangeably use the *s*,*q* notation, and *s*EC + *q*EMC notation), and 2EC + 3EMC. Overall, there is practically no example of *s* + *q* > 5, and typically no solvation environment with *s* + *q* < 4.

**Figure 2:**
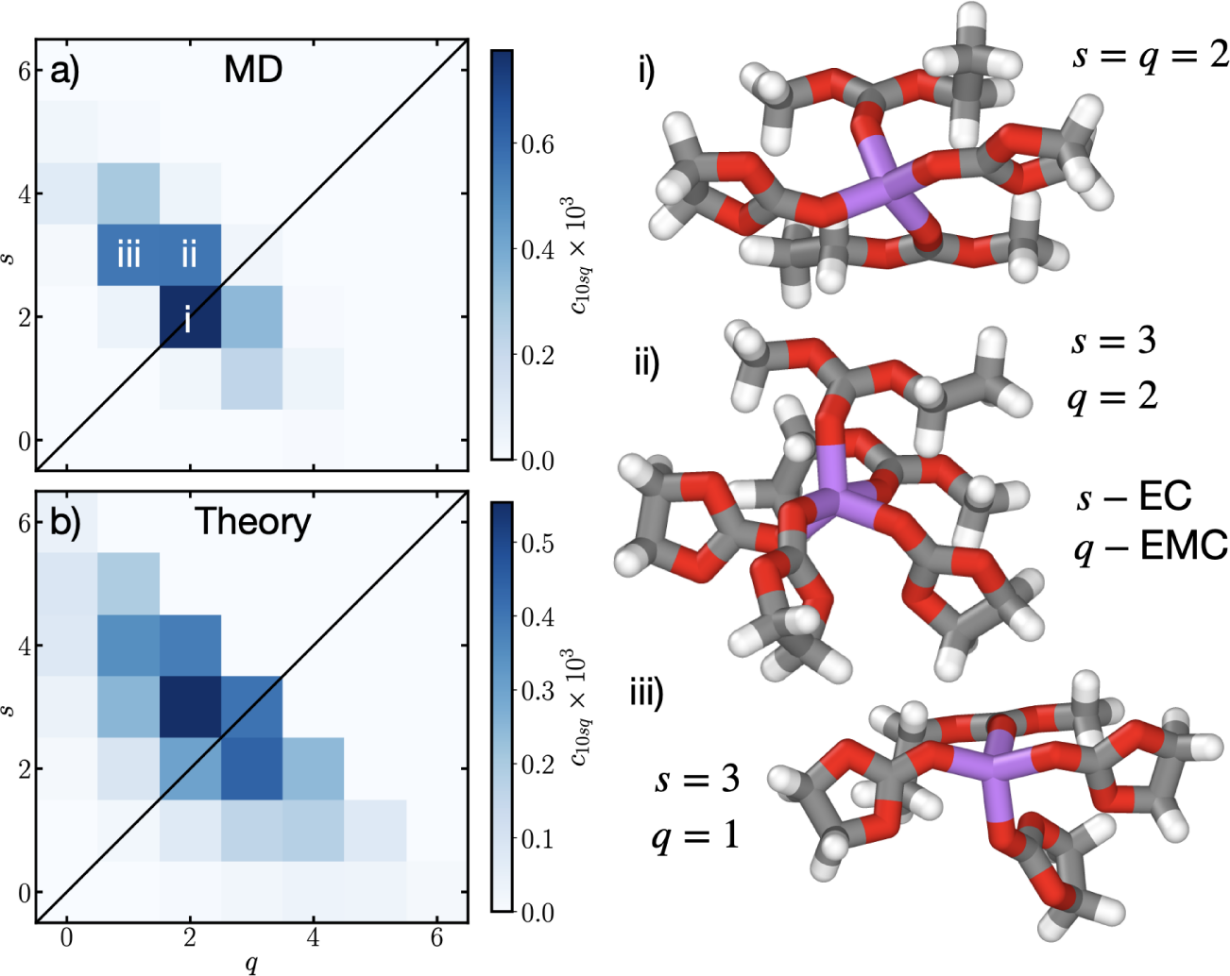
Solvation distributions, *c*_10*sq*_, of Li^+^ in the bulk from MD (a) and theory (b) as a function of the number of coordinating EC (*s*) and EMC (*q*). In (i), (ii) and (iii), example solvation environments for 2EC + 2EMC, 3EC + 2EMC and 3EC + EMC are, respectively, shown, which are the most common solvation environments in MD simulations, as also indicated in (a). These structures were visualized using Ovito.^59^

From these observations, a good choice for *f*_+_ could be 5, with 4, 6 also being reasonable. In the context of WiSE, where there are similar average coordination numbers, it has been found that using *f*_+_ = 4 can lead to better results^52,56^ but the sticky-cation case must then be used. In addition, there are on average 0.87 PF_6_^–^ anions coordinating to Li^+^, bringing the total coordination shell to 5.14. Therefore, with the inclusion of the anions, *f*_+_ = 5 in the sticky-cation approximation and *f*_+_ = 6 in the full theory would be possible; without the role of anions *f*_+_ = 5 for the full solvation distribution or *f*_+_ = 4 for the sticky-cation solvation distribution. These different approaches are displayed in the Supporting Information, with the difference between the theory and MD simulations being quantified.

In Figure 2b, we compare the full theory cluster distribution with *f*_+_ = 6, plotted using eq 1 and the association constants λ_*x*_ = 116.68 and λ_*y*_ = 30.31. As can be seen, the most probable solvation structure involves 3EC + 2EMC, with the next most probable solvation environments containing 2EC + EMC, 3EC + 3EMC and 4EC + 2EMC. While the relative probabilities of these solvation environments do not exactly match the MD simulations, and moreover, the absolute values are slightly different, the overall trend of more EC in the solvation shell compared to EMC is captured. In the Supporting Information, the other example theory comparisons are made, where better agreement in terms of relative solvation distributions are found, but worse quantitative agreement is obtained, where errors in these solvation distributions are shown. Despite there being more EMC in the electrolyte, the ×4 larger association constant between Li–EC compared to Li–EMC results in EC slightly dominating the solvation shell. The disagreement between MD-Theory could be for a number of reasons, such as the MD simulation ensemble averages not being completely converged, or some assumptions of the theory breaking down, such as loop formation or higher-order interactions.

To test if the origin of the disagreement could be from loop formation in the clusters, we computed the cluster bond density (CBD), which is the number of associations in an aggregate over the number of species. In the Cayley-tree limit, the CBD is known to be (*l* + *m* + *s* + *q* – 1)/(*l* + *m* + *s* + *q*) for the electrolyte studied in the main text, with values of CBD being higher than this limit if loops exist. We find that practically all aggregates adhere to the Cayley tree limit, as seen in the Supporting Information, which demonstrates this cannot be a source of disagreement between the MD and Theory. Thus, the assumption of Cayley tree clusters is a very good approximation, and the source of the error must either reside with the MD simulations, or higher order interactions not accounted for in the theory.

### Diffuse EDL

Next, we turn to studying how the solvation environments change within the diffuse EDL at negative electrodes, which is considered to be not the first 5 Å from the interface, but the next 5 Å. Here we only show the solvation effects of the Li^+^ at the negative electrodes, which is the dominant associations occurring in this electrolyte, and do not analyze ionic aggregation as there are only significant numbers of anions for the smallest surface charges. In the Supporting Information, we show additional results for the ionic associations, and solvation and ionic associations at positive electrodes too. In Figure 3 the left column shows the MD results for *c*_10*sq*_ and the right column shows the corresponding theory (calculated with the same method as the bulk). Each row in Figure 3 is a different surface charge, starting from −0.4 *e* nm^–2^ in the top row, to −0.6 *e* nm^–2^ in the middle, to −0.8 *e* nm^–2^ in the bottom row.

**Figure 3:**
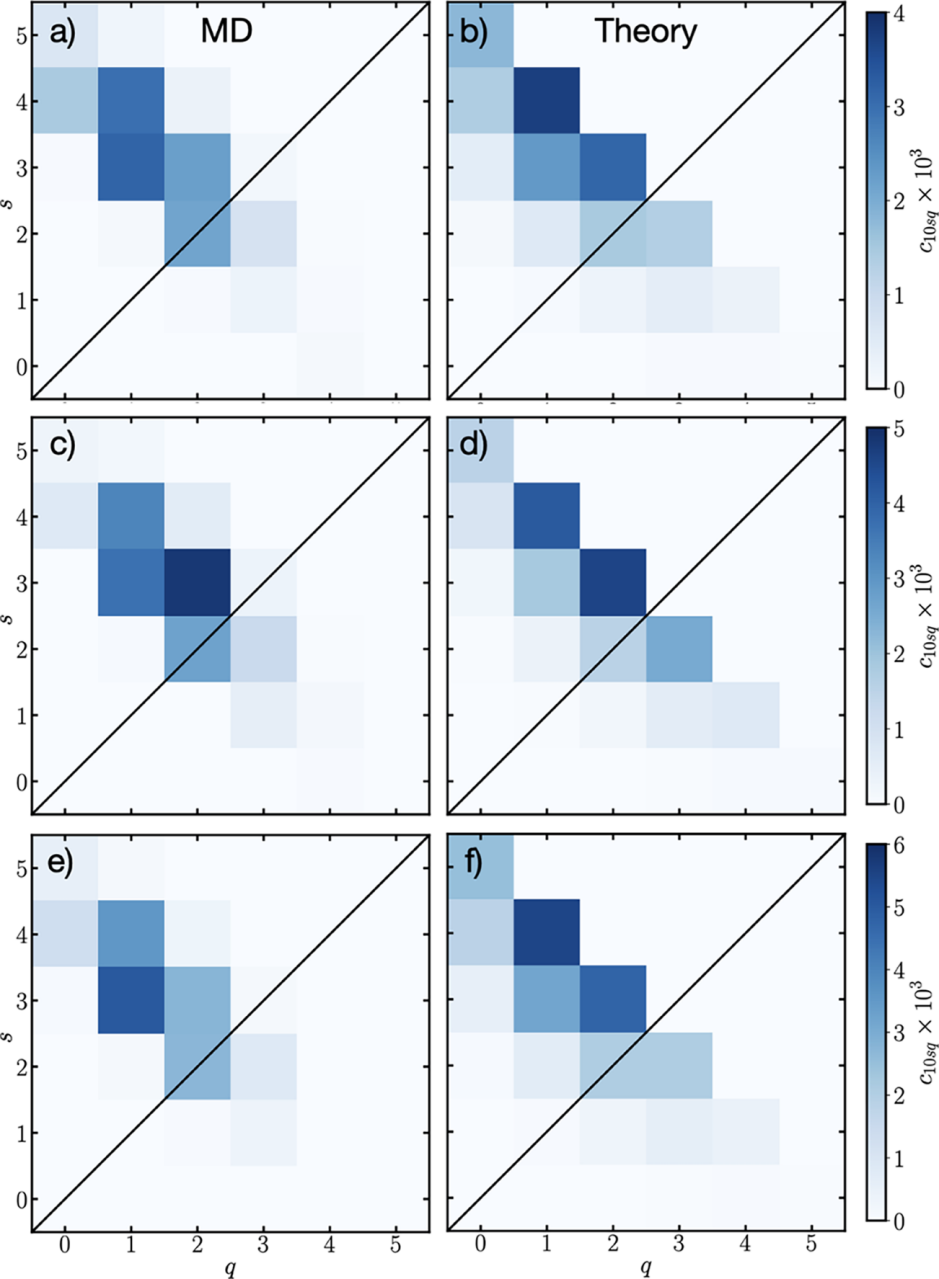
Solvation distributions, *c*_10*sq*_, of Li^+^ in the diffuse EDL from MD [(a), (c), (e)] and theory [(b), (d), (f)] as a function of the number of coordinating EC (*s*) and EMC (*q*) at, respectively, surface charges of −0.4, −0.6 and −0.8 *e* nm^–2^.

For the MD results at −0.4 *e* nm^–2^, as seen in Figure 3a, the solvation structures are fairly similar to the bulk, albeit with larger concentrations of Li^+^ solvation environments owing to the reduced anion concentration. The most probable solvation environment is 3EC + EMC, with 4EC + EMC being the next most probable. The corresponding theory calculation for *c*_10*sq*_, using the association constants calculated from MD using *f*_+_ = 5, displayed in Table 1, is seen in Figure 3b. Clearly, there is a reasonable qualitative match with the MD, even though the exact ordering of the most probable solvation environments are not identical. The theory predicts 4EC + EMC to be the most likely, with 3EC + 2EMC the next most probable. In the Supporting Information, we quantify the difference between the theory and MD simulation solvation distributions.

**Table 1:** Summary of Association Constant Ratios and Mole Fraction Ratios for the EC and EMC Solvents for the Diffuse EDL at the Indicated Surface Charges

σ/*e* nm^–2^	λ_*x*_/λ_*y*_	*x*_*x*_/*x*_*y*_	*p*_+*x*_
–0.4	3.07	1.41	0.64
–0.6	4.40	1.00	0.60
–0.8	6.50	1.50	0.63

At the more negative surface charge of −0.6 *e* nm^–2^, displayed in Figure 3c, *c*_10*sq*_ is again relatively similar to the bulk. In this case, the most probable solvation structure is 3EC + 2EMC. The theory calculation is shown in Figure 3d, where it also predicts that 3EC + 2EMC is the most probable solvation environment, and it also predicts a similar distribution of solvation environments.

Finally, for a surface charge of −0.8 *e* nm^–2^, shown in Figure 3e for MD, the solvation distribution is again relatively similar to the bulk. In this case the most probable solvation environment is 3EC + EMC, with 4EC + EMC being likely too. In Figure 3f the corresponding theory is shown, where we find the most probable solvation environment to be 4EC + EMC. Again, there is reasonable agreement for the spread of solvation environments.

Overall, the solvation environments in the diffuse EDL are similar to those in the bulk, with the theory matching reasonably well against the MD simulations with *f*_+_ = 5, where we also quantify the difference between the theory and MD simulations solvation distributions in the Supporting Information. As seen in Table 1, the ratio of the association constants and molar ratio is displayed for each surface charge. With more negative surface charge, λ_*x*_/λ_*y*_ increases slightly over the bulk value of 3.85, although not substantially. Moreover, the molar ratio of EC relative to EMC is now increased over the bulk value of ∼0.65, reflecting its preferred interaction with the electrostatic fields because of its larger dipole moment, but the association probability between Li^+^–EC is practically constant.

### Helmholtz Layer

Having demonstrated that the theory works well in the bulk and diffuse EDL, as previously found for other electrolytes,^56^ in this section we turn to investigate the solvation environments of Li^+^ in the Helmholtz layer of the anode, which corresponds to the first 5 Å next to the interface. As there are only co-ions present for −0.4 *e* nm^–2^, the only possible effects to study are the solvation environments of Li^+^ cations.

In Figure 4a we show the analysis for the Helmholtz layer for a surface charge of −0.4 *e* nm^–2^ from MD simulations. Similar to the bulk, we find that the most probable environment is 2EC + 2EMC. However, there is practically no solvation structures with *s* + *q* > 4, and very little with *s* + *q* < 4. This is in contrast to the bulk and diffuse EDL cases, when there were significant 5-coordinated Li^+^, and a larger distribution of *s* + *q*.

**Figure 4:**
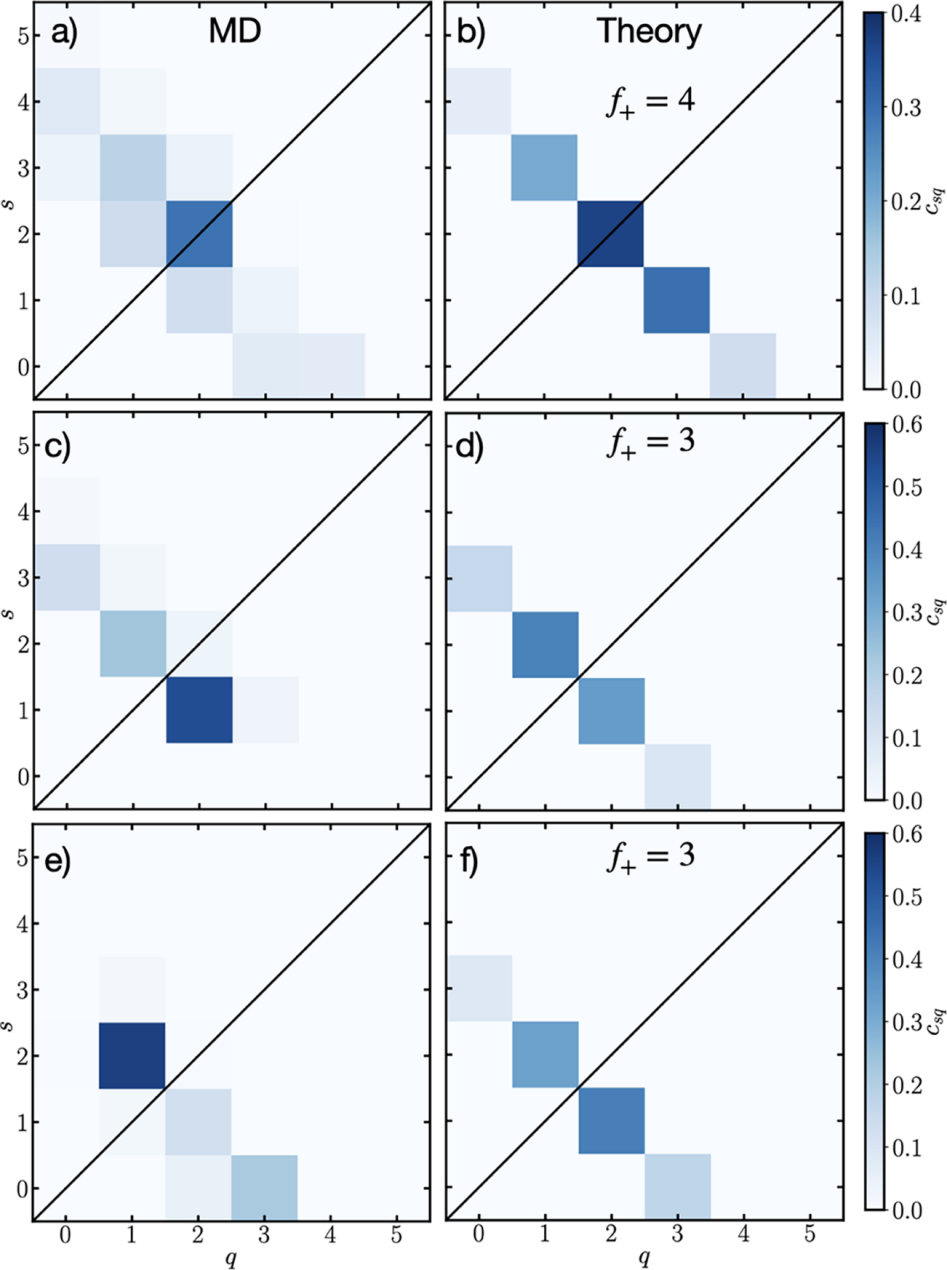
Solvation distributions, *c*_10*sq*_, of Li^+^ in the Helmholtz layer from MD [(a), (c), (e)] and theory [(b), (d), (f)] as a function of the number of coordinating EC (*s*) and EMC (*q*) at, respectively, surface charges of −0.4, −0.6 and −0.8 *e* nm^–2^.

Therefore, it appears that the solvation environment of Li^+^ is behaving in a sticky-way in the Helmholtz layer with *f*_+_ = 3–4, which motivates us to compare the sticky-solvation theory against the MD simulations. In the Supporting Information we compare the MD results against the nonsticky case and demonstrate a worse comparison. Using 1 = *p*_+*x*_ + *p*_+*y*_ (normalized in MD such that this is true) and *f*_+_ = *s* + *q*, we can arrive at

10 $${\mathrm{c̅}}_{sq}=\frac{{\mathrm{c̅}}_{10sq}}{{\mathrm{\phi ̅}}_{+}}=\frac{f_{+}!}{s!\left(f_{+}-s\right)!}{\mathrm{p̅}}_{+x}^{s}{\left(1-{\mathrm{p̅}}_{+x}\right)}^{f_{+}-s}$$

which is simply a binomial distribution for the solvation environments. Hence, the most common solvation environment will be the mode of the binomial distribution with parameters *f*_+_ and *p̅*_+*x*_, explicitly shown in the Supporting Information. The values of *p̅*_+*x*/*y*_ are computed from MD simulations (using the ensemble average coordination numbers) and input into the theory. These are also be used to calculate the ratio of the association constants *λ̅*_*x*_/*λ̅*_*y*_. In Figure 4b we show the theory for the −0.4 *e* nm^–2^ case with *f*_+_ = 4, which clearly agrees well with the MD simulations, with the error between the theory and MD simulation solvation distribution being shown in the Supporting Information.

The MD results for the −0.6 *e* nm^–2^ are shown in Figure 4c. We find that the most probable solvation environment is EC + 2EMC, with 2EC + EMC and 3EC also being possible, but practically no other solvation environment. Therefore, for this surface charge, a better functionality would be *f*_+_ = 3. In Figure 4d we show the corresponding theory plot using *f*_+_ = 3, which agrees reasonably well with the MD simulations. The most probable solvation environment is 2EC + EMC, but the EC + 2EMC is a similar probability.

Finally, for the most negative surface charge results for MD simulations can be found in Figure 4e. Here we find the most probable solvation environment to be 2EC + EMC, with the next most likely being 3EMC. Again, a functionality of *f*_+_ = 3 appears to be a natural choice. In Figure 4f we show the corresponding theory plot, which predicts EC + 2EMC to be the most likely, with 2EC + EMC to be the next most likely.

Overall, the agreement is reasonable between the theory and MD simulations, and these results demonstrate that a reduced functionality works well to describe the solvation environments in the Helmholtz layer, with the difference between the MD simulations and theory solvation distributions being shown in the Supporting Information. This is perhaps not surprising, as the Li^+^ will interact strongly with a charged interface, and block at least one association site of Li^+^. Therefore, when constructing a theory for the Helmholtz layer, we must not use the same functionality in all space, but must reduce it at the interface, meaning that *f*_+_ also becomes an EDL quantity. In the Supporting Information, we more explicitly demonstrate that using *f*_+_ = 5, as in the bulk/diffuse EDL, and only changing the association constant does not provide a satisfactory match with the MD simulations.

In Table 2 we display the ratio of the association constants, λ_*x*_/λ_*y*_, and the molar ratio of the solvents. In contrast to the diffuse EDL, we find λ_*x*_/λ_*y*_ is reduced by more than an order of magnitude at the interface. Note that λ_*x*_/λ_*y*_ does explicitly depend on *f*_+_, but only weakly so through the mass action laws. Therefore, this large reduction is not expected from this change small change in *f*_+_, but we anticipate it is from another source. It can also be seen that the molar ratio of EC is much larger than the bulk, but it appears to saturate near 4× its bulk value.

**Table 2:** Summary of Association Constant Ratios and Mole Fraction Ratios for the EC and EMC Solvents for the Helmholtz Layer at the Indicated Surface Charges

σ/*e* nm^–2^	λ_*x*_/λ_*y*_	*x*_*x*_/*x*_*y*_	*p*_+*x*_
–0.4	0.13	2.75	0.50
–0.6	0.11	4.21	0.52
–0.8	4.44 × 10^–4^	4.24	0.45

As found by Markiewitz et al.^56^ for WiSE (from theory and MD simulations), the λ_*i*_ between Li^+^ and solvents can vary in the EDL if the solvents have a significant dipole moment and can be described as a fluctuating Langevin dipole in the free state. As the dipole moment of EC is much larger than EMC, we would expect λ_*x*_/λ_*y*_ to decrease with increasing electric field, and for the amount of EC to increase relative to EMC as it has a large dipole moment, it will be energetically favorable for it to reside in the larger electric fields. These observations are included in a new theory of the Helmholtz layer, which is outlined in the Supporting Information in detail. In the following section, we present a simplified analysis of this theory.

### Helmholtz Layer Solvation from Bulk Solvation

In the Supporting Information we outline in full the new theory for solvation in the Helmholtz layer. To illustrate its important points, we solve a back-of-the-envelope example here, not solving the system of equations in its full complexity. Our aim is to demonstrate some of its trends, without getting into numerical calculations too much. We assume that there are no anions in the Helmholtz layer (observed in MD for moderate negative surface charges), and that the volume fraction of Li^+^ cations is constant (also observed in MD, at least approximately) and we assume the volumes of each solvent are identical, which means that the only changes occurring is from the solvents swapping places. Note we treat the solvents as fluctuating Langevin dipoles when they are free, but not when they are bound to Li^+^. Therefore, the equations which need to be solved only depend on electric field, which we can approximate from the surface charge density of the simulations.

Furthermore, we work with the sticky-cation approximation, such that the solvent distributions are described by eq 10. The association probabilities for which can be calculated from

11 ψ+p+x=ψxpx+=ψy−ψ++λ(ψ++ψx)2(λ−1)−4ψyψ+(λ−1)+[λ(ψx−ψ+)+ψ++ψy]22(λ−1)

and

12 ψ+p+y=ψypy+=ψy+ψ++λ(ψx−ψ+)2(1−λ)−4ψyψ+(λ−1)+[λ(ψx−ψ+)+ψ++ψy]22(1−λ)

which is the solution of the mass action laws for just solvent in the sticky-cation case. Note that a bar is used to denote quantities within the EDL/Helmholtz layer (*ψ̅*_*i*_, *λ̅*, etc.), which are omitted from eqs 11 and 12 for clarity. Here, the ratio of the association constants in the Helmholtz layer is given by

13 $$\mathrm{\lambda ̅}=\frac{\lambda_{x}}{\lambda_{y}}\frac{p_{x}}{p_{y}}\frac{\sinh\left(\beta p_{y}|\nabla \Phi |\right)}{\sinh\left(\beta p_{x}|\nabla \Phi |\right)}$$

where *p*_*x*_ and *p*_*y*_ are the dipole moments of EC and EMC, respectively, and Φ is the electrostatic potential, with −∇Φ being the electric field. As *p*_*x*_ > *p*_*y*_, the ratio of the association constants decreases with increasing electric field, which is a reflection of EC gaining energy from being a freely fluctuating dipole. This was observed previously, as seen in Table 2.

Next, to determine the composition in the Helmholtz layer, we need to know the volume fractions of each solvent. This can be obtained from a Boltzmann closure relation of the solvents and a statement of incompressibility, following ref. 54. Here, we assume the Boltzmann closure takes the form

14 $$\frac{{\mathrm{\phi ̅}}_{0010}}{{\mathrm{\phi ̅}}_{0001}}=\frac{\phi_{0010}}{\phi_{0001}}\frac{p_{y}}{p_{x}}\frac{\sinh\left(\beta p_{x}|\nabla \Phi |\right)}{\sinh\left(\beta p_{y}|\nabla \Phi |\right)}$$

In the Supporting Information, the full set of closure relations are shown, with the surface interaction terms and Lagrange multiplier for asymmetric sizes, but we only investigate the simplified form here. We also take ϕ_*x*_ + ϕ_*y*_ = ϕ_*xy*_, where ϕ_*xy*_ < 1 is the constant volume fraction of solvent.

From substituting eq 13 into the Boltzmann closure relation, we can simplify eq 14 to obtain

15 $$\frac{{\mathrm{\phi ̅}}_{x}{\mathrm{p̅}}_{x+}}{{\mathrm{\phi ̅}}_{y}{\mathrm{p̅}}_{y+}}=\frac{\phi_{x}p_{x+}}{\phi_{y}p_{y+}}$$

which can also be stated as

16 $$\frac{{\mathrm{p̅}}_{+x}}{{\mathrm{p̅}}_{+y}}=\frac{p_{+x}}{p_{+y}}$$

and therefore, *this approximation states that the probability that the solvents are binding to the association sites do not change from the bulk solvation probabilities*. The bulk value computed for *p*_+*x*_ ≈ 0.48, and the values for *p̅*_+*x*_ are shown in Table 2, which can be seen to be close to the bulk value. Therefore, the MD simulations appear to approximately follow this prediction. Reflecting on the solvation distributions in the Helmholtz layer, and also the diffuse EDL, it can be seen that they do not qualitatively change with surface charge, with the only significant change being the change in functionality, which further supports the simple theory findings here.

As the functionality is reduced in the Helmholtz layer, the numbers of coordinated solvent still decrease, but their relative population in the solvation shell does not change. While the *p*_+*x*/*y*_ does not change, at least given the approximations here, eq 15 does not state that ϕ_*x*/*y*_ and *p*_*x*/*y*+_ need to stay constant, but that the ratio of the Γ_*x*/*y*_ values remains the same as the bulk. In fact, we know the volume fractions significantly change, as seen from the large enhancement of free EC in eq 14, and *p*_*x*/*y*+_ must change because of this, but this is compensated in the change in eq 13.

The volume fractions of solvents and *p*_*x*/*y*+_ could be obtained from eq 15 and eqs 11 and 12, while using the incompressibility constraint (1 = ϕ_+_ + ϕ_*x*_ + ϕ_*y*_). To solve this system of equations, we use a ϕ_+_ = 0.015 (approximately what we find in MD), using volume ratios in ref. 32, λ_*x*_/λ_*y*_ = 3.7 (found from the bulk MD section), and for the dipole moments *p*_*x*_ = 5 D^60^ and *p*_*y*_ = 1 D.^61^ Using a dielectric constant of 5 (which excludes contributions from the dipole moments of the solvents^56,62^ ), we find *λ̅*_–0.4_ = 0.277, *λ̅*_–0.6_ = 0.048 and *λ̅*_–0.8_ = 0.008. From solving the system of equations with these parameters, we find *x̅*_*x*_/*x̅*_*y*_|_–0.4_ = 2.42 while using *f*_+_ = 4, and *x̅*_*x*_/*x̅*_*y*_|_–0.6_ = 4.98 and *x̅*_*x*_/*x̅*_*y*_|_–0.8_ = 5.84 from using *f*_+_ = 3. This demonstrates that even though the relative solvation distribution of Li^+^ is not significantly changing in the Helmholtz layer, mainly through the reduced functionality, the amounts of each solvent are significantly changing. While the agreement is not exact against MD simulations, as seen in Table 2, the qualitative agreement is reasonable.

### Solvation of Additional Electrolytes

In the Supporting Information, we further tested the other electrolytes investigated in ref.^14^. Specifically, the ether solvent mixture with 1,3-dioxolane (DOL) and 1,2-dimethoxyethane (DME), was investigated with lithium bis­(trifluoromethanesulfonyl)­imide (LiTFSI). The ether-based solvents typically interact with the Li^+^ less strongly than the carbonate-based electrolytes, which makes the comparison to our theory more challenging. We find that a functionality of 4 is more appropriate here, but when focusing on only the solvation environments (without ionic aggregate), a functionality of 3 might fit the data better. Despite this more difficult electrolyte, we still observe similar trends to the case of EC + EMC in the main text. Specifically, that the dominant effect in the Helmholtz layer is the apparent reduction in the functionality of Li^+^. However, we only find that *p*_+*x*_ remains (approximately) constant at moderate surface charges, with significant deviations from the bulk value being observed for large surface charges. This demonstrates that the assumptions the result in eq 16 are not universal, and that while the bulk solvation environments are a good starting point, the solvation environments in the EDL should still be investigated.

Moreover, in ref. 14 the solvation in the EDL with the additive fluoroethylene carbonate (FEC) was investigated. In the Supporting Information, we also investigated these three solvent cases. Overall, we find the EC + EMC + FEC cases behaves in a similar way to the EC + EMC mixture and DOL + DME + FEC behaves in an analogous way to DOL + DME, and therefore, we will not discuss these cases further here.

### Ionic Aggregation

Thus far, we have focused on the solvation properties of these electrolytes, which is arguably the dominant effect, but the ionic associations are also important for transport properties and inorganic derived SEI components. In the Supporting Information, we have reported some comparison between theory and MD simulations for ionic aggregation effects for the studied electrolytes, with particular focus on Li–PF_6_ EC–EMC. For this particular electrolyte, some ion pairs exists, but hardly any larger aggregates, and overall the theory reasonably reproduces the MD simulations. In addition we show solvation properties of ion pairs, and find reasonable agreement between the theory and MD simulations.

For the LiTFSI–DOL + DME case, the cation–anion interactions are typically more pronounced than the Li–PF_6_ interactions. In the diffuse layer of this electrolyte, we further investigated the ionic aggregation effects, which is shown in the Supporting Information. At moderate surface charges (0.6 *e* nm^–2^), we find that there are some aggregates larger than ion pairs, which typically does not occur for the other studied surface charges. This suggests that ionic aggregation could be enhanced at some moderate voltages, which was previously found by Markiewitz et al. in the context of salt-in-ionic liquids^57,58^ and water-in-salt electrolytes.^56^

### Discussion

Overall, the main effect we observe from thoroughly analyzing the solvation environments in the Helmholtz layer of nonaqueous battery electrolytes is that they (largely) appear to be the same as the bulk solvation environments, but where the number of association sites of Li^+^ is reduced. This is perhaps not surprising, as the interface physically blocks some association sites and interacts with the Li^+^. Moreover, reduced coordination numbers of solvents has been reported in myriad other simulations of nonaqueous battery electrolytes.^11,14,36,37^ However, here we show that the effect is best described through changing the number of available binding sites (and the Li-solvent binding free energy), instead of only changing the Li-solvent interactions.

There are several implications of this observation. As an equilibrium between the Helmholtz layer and bulk must be established, it becomes apparent that even without any applied fields or interactions with the surface, *the electrolyte can become charged from the reduction of functionalities at the interface*. Moreover, it does not appear that it is necessary to establish an equilibrium between the diffuse EDL and the Helmholtz layer, although one could be established, but as both of them are in equilibrium with the bulk, they should both be in equilibrium with each other.

Under certain assumptions, we found that the *cation-solvent association probabilities remain constant in the Helmholtz layer, and moreover, equal to their bulk values*. If these assumptions apply to an electrolyte, it means only the bulk solvation environments need to be investigated, and the Helmholtz layer solvation environments can simply be predicted from the developed theory with a reduced *f*_+_ (to 3–4, from values of 4–6 in the bulk). We found that EC + EMC approximately follows these assumptions, but that DOL + DME did only for small surface charges. Therefore, this observation is not universal, and the assumptions can be broken in real electrolyte systems, which means investigating the EDL of these electrolytes is still necessary to test if this observation holds. Moreover, we found that sometimes the functionality reduces by 1, but it can reduce by 2, depending on surface charge. Therefore, performing EDL simulations of the electrolytes is still important to establish their functionalities in the Helmholtz layer.

The theory developed here is a simple lattice-gas mean-field theory, that accounts for correlations beyond mean-field through the associations between species. While it is not sophisticated, it is analytically tractable and physically interpretable. It does, however, miss some correlations beyond mean-field and struggles with the spatial resolution of species in the EDL, as does any local density approximation. Beyond this local density approximation, some of the other important assumptions of the theory are the assumed Cayley-tree clusters, sticky-cation approximation, treatment of the solvents as fluctuating Langevin dipoles in the free state but frozen in the solvated state, and we assume no further interactions beyond these associations, among some additional smaller approximations. We have proven the Cayley-tree clusters is an extremely good approximation here and the sticky-cation approximation is good for the Helmholtz layer, but the other employed approximations could be resulting in the disagreement between the theory and MD simulations. For further discussion of the limitations of the theory see refs. 54, 56 ,^58^, with future work further investigating these limitations.

Currently, there is not a standardized convention for reporting solvation environments and ionic aggregates. Typically, coordination numbers, some ion pairs and aggregates group together are reported in a nonstandardized way. As discussed in ref. 32, coordination numbers do not provide a unique classification of the associations, and further information is required, such as the cluster bond density. Here we have found it insightful to plot the solvation distributions for free cations as a function of the number of each bound solvent. This provided a natural way to visualize the results, which gave insight into the Helmholtz layer, as well as bulk and diffuse EDL solvation. Therefore, we suggest that the reporting convention outlined in ref. 32 can provide a natural framework to work within to further understand these complex electrolytes. Importantly, this convention can not only be applied to classical MD simulations, but any atomistic simulation method, such as *ab initio* MD, machine learning MD, and the inclusion of quantum nuclear effects (as solvation properties are a statistical thermodynamic property, quantum nuclear effects could change the solvation structures in nontrivial ways, but we still expect the theory developed here to be able to describe those simulations).

Looking forward, the formalism developed here could be extended to a Stern model, where both Helmholtz layer and diffuse EDL are combined in series, and perhaps integrated further. Moreover, the theory could be integrated with microscopic models of electrochemical reaction kinetics, such as coupled ion-electron transfer theory through the solvent reorganization energy^63^ to theoretically investigate possible reactions at interfaces. Finally, the motivation of studying solvation environments in the first monolayer of an electrified interface, i.e., the Helmholtz layer, is to predict the species that may reacting at the interface to form the SEI. Therefore, our theory could be used to predict possible solvation environments in the Helmholtz layer from bulk solvation environments from MD simulations or experiments, and then use these in DFT to determine the reductive stability of these solvation environments, or using them as inputs/biases for reaction networks to predict what could form in the SEI. The presented theory can also be used to estimate conductivity and transference numbers, although these transport properties are quite difficult to accurately predict, and moreover, would only include vehicular transport of species, ignoring all contributions from structural transport.

Here we have demonstrated that parameters for a single electrolyte work well for changes in its composition, and previous work has shown that more systematic changes in composition can be accurately captured. The central parameters that need to be identified for the theory are the functionalities and association constants. Some Li^+^ and Na^+^ electrolytes have been studied so far,^32,51–53,56^ but myriad more electrolytes exist of interest for various applications. In the future, it would be interesting to investigate other electrolytes, identify trends in the association constants between different chemistries, and maybe some day have a library of parameters that could be used without having to perform MD simulations to obtain these parameters. Moreover, these association constants can be used as a key descriptor for describing solvation and ionic association strength, which is more of a natural descriptor than ion pair energies in vacuum, etc., which could be useful for understanding trends in different electrolyte formulations and coming up with design principles.

## Conclusion

In conclusion, from analyzing in detail the solvation environments predicted from molecular dynamics (MD) simulations, we have uncovered desired criteria for developing a theory to describe the solvation structures in the Helmholtz layer. Specifically, we find reduced numbers of solvents binding to the active cations and few ionic aggregates. This inspired the development of a modified theory to describe these solvation environments, which we test against these MD simulations, and found reasonable qualitative agreement. One of the novel predictions of this theory, under certain simplifying assumptions, is that the association probabilities between the cations and solvents remain equal to the bulk values. Moreover, the developed theory can be parametrized from bulk MD data, and the only change that needs to be made is the maximum number of associations a cation can form, as some of its association sites interact with the electrode, and cannot participate in solvation. Overall, we hope this theory will provide a framework for understanding electrolytes with strong specific interactions to electrified interfaces.

## Supplementary Material



## Notes

Derivation for the diffuse electrical double layer theory, Helmholtz layer theory, in addition to a further comparison against molecular dynamics simulations (PDF)
