# Supplementary material for: Theory of Cation Solvation in the Helmholtz Layer of Li-Ion Battery Electrolytes
Source: ACS Appl Energy Mater. 2025 Jun 2;8(12):8376–87. doi: 10.1021/acsaem.5c00883 (PMC12188516; doi:10.1021/acsaem.5c00883)
Supplement: Supplementary file 1 [file ae5c00883_si_001.pdf]

# Supporting Information: Theory of Cation Solvation in the Helmholtz Layer of Li-ion Battery Electrolytes


Zachary A. H. Goodwin,[1,2,*] Daniel M. Markiewitz,[3]
Qisheng Wu,[4] Yue Qi,[4] and Martin Z. Bazant[3,5]

[1]*Department of Materials, University of Oxford, Parks Road, Oxford OX1 3PH, United Kingdom*

[2]*John A. Paulson School of Engineering and Applied Sciences, Harvard University, Cambridge, Massachusetts 02138, United States*

[3]*Department of Chemical Engineering, Massachusetts Institute of Technology, Cambridge, Massachusetts 02139, USA*

[4]*School of Engineering, Brown University, Providence, RI, 02912, USA*

[5]*Department of Mathematics, Massachusetts Institute of Technology, Cambridge, Massachusetts 02139, USA*

[*] zac.goodwin@materials.ox.ac.uk

## I. DIFFUSE ELECTRICAL DOUBLE LAYER THEORY

Here we outline in detail an example of the theory for the diffuse electrical double layer (EDL), such that it can be contrasted with the version for the Helmholtz layer in the next section. Following the work of Refs. 1–3, which we recommend the readers familiarize themselves with first, we assume the free energy, $\mathcal{F}$, functional for a salt containing 3 solvents with dipole moments to be

$$\begin{aligned}\beta\mathcal{F} = &\int_V d\mathbf{r}\left\{-\beta\frac{\epsilon_0\epsilon_r}{2}\left(\nabla\Phi\right)^2 + \beta\rho_e\Phi - \frac{c_{00100}}{v_+}\mathcal{L}_x - \frac{c_{00010}}{v_+}\mathcal{L}_y - \frac{c_{00001}}{v_+}\mathcal{L}_z\right\} \\ &+ \int_V d\mathbf{r}\frac{1}{v_+}\sum_{lmijk}\left(c_{lmijk}\ln\phi_{lmijk} + c_{lmijk}\beta\Delta_{lmijk}\right) \\ &+ \int_V d\mathbf{r}\frac{\Lambda}{v_+}\left\{1 - \sum_{lmijk}(l + \xi_- m + \xi_x i + \xi_y j + \xi_z k)c_{lmijk}\right\}\end{aligned} \tag{S1}$$

Here the first line represents the electrostatic free energy, where $\beta = 1/k_BT$ is inverse thermal energy (with $k_B$ being the Boltzmann constant and $T$ being the temperature), $\epsilon_0$ is the permittivity of free space, and $\epsilon_r$ is the relative permittivity of the electrolyte, $\Phi$ is the electrostatic potential, $-\nabla\Phi$ is the electric field, $\rho_e$ is the charge density, given by $\rho_e = \frac{e}{v_+}(c_+ - c_-)$, with $e$ denoting elemental charge, $c_+ = \sum_{lmijk} lc_{lmijk}$, and $c_- = \sum_{lmijk} mc_{lmijk}$ are the dimensionless concentrations of cations and anions, respectively, with $c_{lmijk}$ denoting the dimensionless clusters of rank $lmijk$, where there are $l$ cations, $m$ anions, and $i$, $j$ and $k$ of the 3 solvents, which are related to their concentrations through similar expressions, $c_x = \sum_{lmijk} ic_{lmijk}$, $c_y = \sum_{lmijk} jc_{lmijk}$,$c_z = \sum_{lmijk} kc_{lmijk}$. Finally, the last 3 terms of the line represent the contributions from the solvents being treated as fluctuating Langevin dipoles

$$\mathcal{L}_x = \ln\left\{\frac{\sinh(\beta p_x|\nabla\Phi|)}{\beta p_x|\nabla\Phi|}\right\}, \tag{S2}$$

where $p_x$ is the dipole moment of the solvent. Here the volume of a lattice site is set to $v_+$, the concentration of free solvents is denoted by $c_{00100}$, $c_{00010}$, $c_{00001}$.

The second line of Eq. (S1) represents the chemical free energy of forming the aggregates/solvation environments. Here $\phi_{lmijk}$ is the volume fraction of the cluster of that rank,

given by $\phi_{lmijk} = (l + \xi_- m + \xi_x i + \xi_y j + \xi_z k)c_{lmijk}$, where $\xi_{-/x/y/z} = v_{-/x/y/z}/v_+$ represents the relative volume occupied by species to the size of the cation. Note that the total volume fraction of each species can be determined from $\phi_- = \xi_- c_-$, for example. Finally, the free energy of forming a cluster of rank $lmijk$ is $\Delta_{lmijk}$, which has two contributions

$$\Delta_{lmijk} = \Delta^{comb}_{lmijk} + \Delta^{bind}_{lmijk}, \tag{S3}$$

where the first term is the combinatorial contribution from the number of ways of arranging the ions in each cluster, and the second term is the binding free energy of each cluster. In the context of polymers, Stockmayer solved the combinatorial entropy for Cayley tree associations

$$\Delta^{comb}_{lmijk} = k_B T \ln\{f_+^l f_-^m W_{lmijk}\}, \tag{S4}$$

where $f_+$ and $f_-$ are the functionalities of the cations and anions, respectively, and $W_{lmijk}$ is

$$W_{lmijk} = \frac{(f_+ l - l)!(f_- m - m)!}{l!m!i!j!k!(f_+ l - l - m - i - j - k + 1)!(f_- m - m - l + 1)!}. \tag{S5}$$

Moreover, the binding free energy is simply given by

$$\Delta^{bind}_{lmijk} = (l + m - 1)\Delta f_- + i\Delta f_x + j\Delta f_y + k\Delta f_z, \tag{S6}$$

provided there is at least one cation in the cluster, owing to the assumption of Cayley tree clusters.

The third line of Eq. (S1) represents the enforcement of incompressibility through a Lagrange multiplier, $\Lambda$. This was introduced by Markiewitz *et al.* [1, 2], and is essential when considering asymmetric systems.

Here we consider our system in the pre-gel regime. This is a reasonable approximation, as battery electrolytes are typically not concentrated enough for large aggregates and ionic networks to form.

By taking the functional derivative of our free energy with respect to $c_{lmijk}$, we can obtain the chemical potential of each cluster

$$\beta\mu_{lmijk} = (l - m)\beta e\Phi + 1 + \ln(\phi_{lmijk}) + \beta\Delta_{lmijk} - (l + \xi_- m + \xi_x i + \xi_y j + \xi_z k)\Lambda. \tag{S7}$$

In the case of free solvents, we obtain, for example

$$\beta\mu_{00100} = 1 + \ln(\phi_{00100}) - \mathcal{L}_x + \beta\Delta_{00100} - \xi_x\Lambda \tag{S8}$$

From establishing the cluster equilibrium

$$l\mu_{10000} + m\mu_{01000} + i\mu_{00100} + j\mu_{00010} + k\mu_{00001} = \mu_{lmijk}, \tag{S9}$$

we can obtain an expression for the concentrations of all ranks of clusters

$$c_{lmijk} = \frac{W_{lmijk}}{\lambda_-}(\lambda_-\psi_{10000})^l(\lambda_-\psi_{01000})^m(\lambda_x\psi_{00100})^i(\lambda_y\psi_{00010})^j(\lambda_z\psi_{00001})^k \tag{S10}$$

where $\psi_{10000} = f_+\phi_{10000}$ and $\psi_{01000} = f_-\phi_{01000}/\xi_-$ are the number of free cation and free anion association sites per lattice site, respectively, and similar for the solvents $\psi_{00100} = \phi_{00100}/\xi_x$, $\psi_{00010} = \phi_{00010}/\xi_y$, and $\psi_{00001} = \phi_{00001}/\xi_z$. Here, the $\lambda_{-/x/y/z}$ represents the the *association constant* between cations and each species, given by $\lambda_{-/x/y/z} = \exp\{-\beta\Delta f_{-/x/y/z}\}$. This is one of the most important parameters of the theory, which is physically interpretable.

Note Eq. (S10), the cluster distribution, holds in the bulk and in the EDL, but where the volume fractions and association constants are replaced by their respective EDL quantities [3]. To be more explicit from hereon out, EDL quantities are denoted with a bar. For example, from the theory we find the $\lambda_{x/y/z}$ are not constant in the EDL, but vary because of the changes in energy of free species relative to clusters, as seen through

$$\bar{\lambda}_x = \lambda_x \exp(-\mathcal{L}_x). \tag{S11}$$

Analogous expressions exist for the other solvents, and the cation-anion $\lambda$ constant is found to remain field-independent.

In Eq. (S10), the concentrations of each cluster of rank $lmijk$ is determined from the association constants and the concentrations of all the free species, but these are the quantities that we wanted to predict from the theory, not be inputs to the theory. To allow us to solve for the cluster distribution, we need to introduce the idea of association probabilities, conservation of associations and the law of mass action on associations. As $\phi_{10000}$ and $\phi_{01000}$ are, in principle, inaccessible *a priori*. Instead, it is natural to express the cluster distribution in terms of the overall volume fractions of each species, $\phi_i$, which is an experimentally/computationally controllable parameter. This connection is established by

introducing ion association probabilities, $p_{ij}$, which is the probability that an association site of species $i$ is bound to species $j$. Therefore, the volume fraction of free cations can be written as $\phi_{10000} = \phi_+(1-p_{+-}-p_{+x}-p_{+y}-p_{+z})^{f_+}$ and free anions as $\phi_{01} = \phi_-(1-p_{-+})^{f_-}$, and a free solvent $\phi_{00100} = \phi_x(1-p_{x+})$, for example.

The association probabilities can be determined through the conservation of associations and a mass action law between open and occupied association sites. The conservation of associations is given by

$$p_{+-}\psi_+ = p_{-+}\psi_- = \zeta_+, \tag{S12}$$

$$p_{+x}\psi_+ = p_{x+}\psi_x = \zeta_x, \tag{S13}$$

$$p_{+y}\psi_+ = p_{y+}\psi_y = \zeta_y, \tag{S14}$$

$$p_{+z}\psi_+ = p_{z+}\psi_z = \zeta_z. \tag{S15}$$

The mass action laws between open and occupied association sites is

$$\lambda_+\zeta_+ = \frac{p_{+-}p_{-+}}{(1-p_{+-}-p_{+x}-p_{+y}-p_{+z})(1-p_{-+})}, \tag{S16}$$

$$\lambda_+\zeta_x = \frac{p_{+x}p_{x+}}{(1-p_{+-}-p_{+x}-p_{+y}-p_{+z})(1-p_{x+})}, \tag{S17}$$

$$\lambda_+\zeta_y = \frac{p_{+y}p_{y+}}{(1-p_{+-}-p_{+x}-p_{+y}-p_{+z})(1-p_{y+})}, \tag{S18}$$

$$\lambda_+\zeta_z = \frac{p_{+z}p_{z+}}{(1-p_{+-}-p_{+x}-p_{+y}-p_{+z})(1-p_{z+})}. \tag{S19}$$

Here $\zeta_+ = \psi_-p_{-+} = \psi_+p_{+-}$ is the dimensionless concentration of associations (per lattice site), $\psi_+ = f_+\phi_+$ and $\psi_- = f_-\phi_-/\xi_-$ are the number of cation and anion association sites per lattice site, respectively, and similar for the solvents $\psi_x = \phi_x/\xi_x$, $\psi_y = \phi_y/\xi_y$, and $\psi_z = \phi_z/\xi_z$.

In the sticky-cation approximation, i.e., when all cation association sites are occupied, and if we assume no cation-anion binding, we have $f_+ = s+q+p$ and $1 = p_{+x}+p_{+y}+p_{+z}$.

Using these relationships, the mass-cation laws and the conservation of associations, we find the solvation distribution to be

$$c_{sqp} = \frac{c_{10sqp}}{\phi_+} = \frac{f_+!}{s!q!p!} p_{+x}^s p_{+y}^q p_{+z}^p. \tag{S20}$$

This states that the solvation distributions of a single cation is simply a multinomial. The two-solvent case shown in the main text can be obtained from taking the limiting case of $p = 0$. In this two-species limit, the most common number of solvent $s$ in the solvation environment is

$$\text{Most likely s} = \begin{cases} \lfloor (f_+ + 1)p_{+x} \rfloor & (f_+ + 1)p_{+x} = 0 \text{ or } \notin \mathbf{Z} \\ (f_+ + 1)p_{+x} \,\&\, (f_+ + 1)p_{+x} - 1 & (f_+ + 1)p_{+x} \in \{1, ..., f_+\} \\ f_+ & (f_+ + 1)p_{+x} = f_+ + 1 \end{cases} \tag{S21}$$

Unfortunately, the most probable state cannot be as cleanly derived for the general solvent case as the states are multinomially distributed, which does not have a simple definition for the most likely state.

To establish the bulk-EDL equilibrium, as introduced by Goodwin *et al.* [3], we set the chemical equilibrium between the free species in the bulk and the EDL. For each species, this is seen as

$$\bar{\phi}_{10000} = \phi_{10000} \exp(-\beta e \Phi + \Lambda), \tag{S22}$$

$$\bar{\phi}_{01000} = \phi_{01000} \exp(\beta e \Phi + \xi_- \Lambda), \tag{S23}$$

$$\bar{\phi}_{00100} = \phi_{00100} \exp(\mathcal{L}_x + \xi_x \Lambda), \tag{S24}$$

$$\bar{\phi}_{00010} = \phi_{00010} \exp(\mathcal{L}_y + \xi_y \Lambda), \tag{S25}$$

$$\bar{\phi}_{00001} = \phi_{00001} \exp(\mathcal{L}_z + \xi_z \Lambda). \tag{S26}$$

In the EDL, however, we do not know the volume fractions and the association probabilities, which we need to solve for. In total, there are 5 unknowns from the volume fractions, and 8 unknown association probabilities. In addition, the Lagrange multiplier, $\Lambda$, needs to be found. In total, there are 14 unknowns which need to be determined. From the 4 mass action laws, 4 conservations of associations, 5 bulk-EDL equilibrium's, and the incompressibility constraint from the Lagrange multiplier, we have 14 equations which need to be simultaneously solved. This then establishes the concentration dependence as a function of

the electrostatic potential and electric field. In the case of a sticky-cation approximation being used, a similar procedure is used, with details in Refs. 1, 2.

To predict the behavior of this electrolyte in the EDL, we can derive our modified Poisson-Boltzmann equation, by taking the functional derivative of the free energy with respect to the electrostatic potential

$$\nabla \cdot (\epsilon \nabla \Phi) = -\bar{\rho}_e, \tag{S27}$$

where

$$\epsilon = \epsilon_0 \epsilon_r + p_x \frac{\bar{c}_{00100}}{v_+} \frac{L(\beta p_x \nabla \Phi)}{\nabla \Phi} + p_y \frac{\bar{c}_{00010}}{v_+} \frac{L(\beta p_y \nabla \Phi)}{\nabla \Phi} + p_z \frac{\bar{c}_{00001}}{v_+} \frac{L(\beta p_z \nabla \Phi)}{\nabla \Phi} \tag{S28}$$

is the dielectric function, with $L(x) = \coth(x) - 1/x$ being the Langevin function. First, maps between the concentrations of species, and electrostatic potential and electric field are established, which are then used to numerically solve the modified Poisson Boltzmann equation [1, 2].

## II. HELMHOLTZ LAYER THEORY

Following the previous section, we develop a version of the theory which is more specific to the Helmholtz layer, i.e., a layer of the electrode right next to the electrode. We assume that the free energy functional takes the form

$$\beta\mathcal{F} = \int_V d\mathbf{r} - \beta\frac{\epsilon_0\epsilon_r}{2}\left(\nabla\Phi\right)^2 + \beta\rho_e\Phi - \frac{c_{00100}}{v_+}\mathcal{L}_x - \frac{c_{00010}}{v_+}\mathcal{L}_y - \frac{c_{00001}}{v_+}\mathcal{L}_z$$
$$+ \int_V d\mathbf{r}\frac{1}{v_+}\sum_{lmijk}\left(c_{lmijk}\ln\phi_{lmijk} + c_{lmijk}\beta\Delta_{lmijk}\right) + \sum_{i'} W_{i'}c_{i'}$$
$$+ \int_V d\mathbf{r}\frac{1}{v_+}\Lambda\left(1 - \sum_{lmijk}(l + \xi_- m + \xi_x i + \xi_y j + \xi_z k)c_{lmijk}\right). \tag{S29}$$

Overall, this is a similar functional as the diffuse EDL, but where there is an additional term on the second line from the interactions of species with the electrode, and the integral over volume is only a small region of electrolyte adjacent to the electrode. The $W_{i'}$ terms represent the interaction strength of each species with the electrode, which is assumed to interact with each species, irrespective of their participation in clusters. The addition of this surface term does not modify the cluster distribution, or the mass action laws or conservation of associations. If we considered interactions to depend on the associations of species, modifications of these quantities might be expected, but we do not explore this case here. For quantities within the Helmholtz layer, we use a tilde to be more explicit that these are quantities in this region.

From taking the functional derivative with respect to $\tilde{c}_{lmijk}$, where a tilde is used to denote Helmholtz layer quantities, we obtain the electrochemical potential of the clusters

$$\beta\tilde{\mu}_{lmijk} = (l - m)\beta e\Phi + 1 + \ln(\tilde{\phi}_{lmijk}) + \beta\Delta_{lmijk} + W_+ l + W_- m + W_x i + W_y j + W_z k$$
$$- (l + \xi_- m + \xi_x i + \xi_y j + \xi_z k)\Lambda, \tag{S30}$$

and for the free solvent species, we have

$$\beta\tilde{\mu}_{00100} = 1 + \ln(\tilde{\phi}_{00100}) - \mathcal{L}_x + W_x + \beta\Delta_{00100} - \xi_x\Lambda. \tag{S31}$$

We can then set up an equilibrium between free species in the Helmholtz layer and the species in the bulk, to obtain the closure relations of the species

$$\tilde{\phi}_{10000} = \phi_{10000}\exp(-\beta e\Phi - W_+ + \Lambda), \tag{S32}$$

$$\tilde{\phi}_{01000} = \phi_{01000}\exp(\beta e\Phi - W_{-} + \xi_{-}\Lambda), \tag{S33}$$

$$\tilde{\phi}_{00100} = \phi_{00100}\exp(\mathcal{L}_x - W_x + \xi_x\Lambda), \tag{S34}$$

$$\tilde{\phi}_{00010} = \phi_{00010}\exp(\mathcal{L}_y - W_y + \xi_y\Lambda), \tag{S35}$$

$$\tilde{\phi}_{00001} = \phi_{00001}\exp(\mathcal{L}_z - W_z + \xi_z\Lambda). \tag{S36}$$

The solution to the system of equations can be obtained in an analogous way.

It should be noted that a main conclusion from the main text (and as further shown for other electrolytes later) is that the cations appear to behave as if they have a smaller functionality in the Helmholtz layer than in the bulk and diffuse EDL. This can be rationalized through these species interacting with the electrode and blocking association sites. The implication of this, however, is that the bulk-EDL equilibrium's are between the free species, at least in we follow Ref. 3, which means that in the absence of applied electrostatic fields, the concentrations of free species is modified by the surface interaction terms and *the differing functionalities from the Helmholtz layer and the bulk.* Therefore, the volume fractions of species can differ in the Helmholtz layer, causing electroneutrality breakdown, for example, even in the absence of applied fields.

As discussed in the main text, let us investigate a simplified case, but for a 3-solvent case. We again assume no anions, a constant cation volume fraction, no surface interactions and the same sizes for all solvents, and the sticky-cation approximation ($1 = p_{+x} + p_{+y} + p_{+z}$), we could write down the Boltzmann closure relationships as

$$\frac{\tilde{\phi}_{00100}}{\tilde{\phi}_{00010}} = \frac{\phi_{00100}}{\phi_{00010}}\frac{p_y}{p_x}\frac{\sinh(\beta p_x|\nabla\Phi|)}{\sinh(\beta p_y|\nabla\Phi|)}, \tag{S37}$$

and

$$\frac{\tilde{\phi}_{00100}}{\tilde{\phi}_{00001}} = \frac{\phi_{00100}}{\phi_{00001}}\frac{p_z}{p_x}\frac{\sinh(\beta p_x|\nabla\Phi|)}{\sinh(\beta p_z|\nabla\Phi|)}. \tag{S38}$$

In the stick-cation approximation, we must divide the mass-action laws to remove the singularities. This is

$$\frac{\tilde{\lambda}_x}{\tilde{\lambda}_y} = \frac{\tilde{p}_{x+}(1-\tilde{p}_{y+})}{\tilde{p}_{y+}(1-\tilde{p}_{x+})} = \frac{\lambda_x}{\lambda_y}\frac{p_x}{p_y}\frac{\sinh(\beta p_y|\nabla\Phi|)}{\sinh(\beta p_x|\nabla\Phi|)}, \tag{S39}$$

and

$$\frac{\tilde{\lambda}_x}{\tilde{\lambda}_z} = \frac{\tilde{p}_{x+}(1-\tilde{p}_{z+})}{\tilde{p}_{z+}(1-\tilde{p}_{x+})} = \frac{\lambda_x}{\lambda_z}\frac{p_x}{p_z}\frac{\sinh(\beta p_z|\nabla\Phi|)}{\sinh(\beta p_x|\nabla\Phi|)}. \tag{S40}$$

Again, these expressions can be inserted into the Boltzmann closure relationships and simplified to arrive at

$$\frac{\bar{\phi}_x\bar{p}_{x+}}{\bar{\phi}_y\bar{p}_{y+}} = \frac{\phi_x p_{x+}}{\phi_y p_{y+}}, \tag{S41}$$

and

$$\frac{\bar{\phi}_x\bar{p}_{x+}}{\bar{\phi}_z\bar{p}_{z+}} = \frac{\phi_x p_{x+}}{\phi_z p_{z+}}. \tag{S42}$$

These are analogous expressions to those in the main text. Again the conservation of associations can be employed to show that $\tilde{p}_{+x/y/z} = p_{+x/y/z}$. To solve for how the volume fractions change in the Helmholtz layer, we solve the above equations with the mass action laws and conservation of associations. Note that the choice of Boltzmann closure relationships and sticky-cation equations was arbitrary, with other combinations being possible, but choosing them to be consistent helps simplify the resulting equations, and shouldn't change the final results. Finally, we note that these equations would also hold in the diffuse EDL, provided the assumptions hold (a constant cation volume fraction and no anions generally does not hold, which is why we have not interpreted these equations in the context of the diffuse EDL).

## III. FURTHER COMPARISON TO MOLECULAR DYNAMICS SIMULATIONS

### A. Cluster bond density

The cluster bond density (CBD) is the number of associations/bonds in a cluster over the number of species in that cluster

$$CBD = \frac{\#\text{Number of Bonds}}{\#\text{ Number of species}}. \tag{S43}$$

In the Cayley tree limit, it is known to be

$$CBD_{Cayley} = \frac{\sum j - 1}{\sum j}, \tag{S44}$$

where the sum over $j = l, m, s, q, p$ is for the number of each species, and it is known how many associations there are based on the number of species. Therefore, if the MD computed CBD matches well this Cayley tree limit, this is a good assumption of our theory.

In Fig. S1 we show the computed CBD from the MD simulations to test the Cayley tree approximation of our clusters. As can be seen, almost all computed clusters adhere to this limit, with only a few examples demonstrating clusters with 1-3 loops. Specifically, for EC-EMC, there's only one type of a cluster with a single loop, meaning the approximation is excellent, while DOL+DME has a number of clusters with a single loop, and several with more than one. The EC+EMC+FEC example has a few more looped clusters than EC+EMC, but the DOL+DME+FEC has no clusters with loops. Overall, the Cayley tree limit is well held, with only a few clusters with ∼10 species having a few loops.

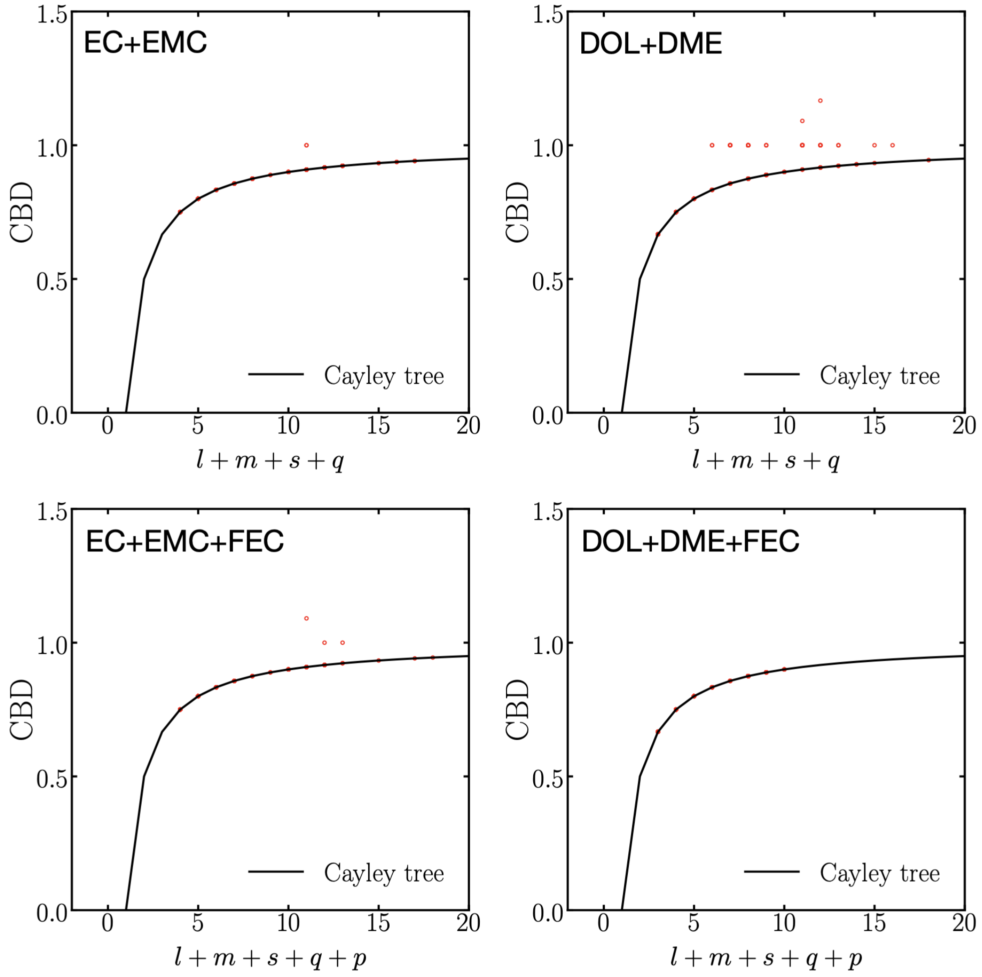


FIG. S1. Cluster bond density (CBD) as a function of the number of species in each cluster, for the various studied electrolytes in the bulk region. The Cayley tree limit is indicated.

## B. EC+EMC

### 1. *Bulk*

In Fig. S2 we show further examples of different comparisons of the theory (left) for the bulk solvation distribution, with the accompanying difference between the theory and MD simulations (right). In the top row, we show the case of $f_+ = 6$ for the full theory, i.e., accounting for ionic aggregates too. The solvation distribution is discussed in the main text. We see there is some large error (55% out from MD) in the predicted concentration of 2EC+2EMC, which the theory is underestimating, and some smaller errors of the other solvation structures surrounding 3EC+2EMC.

For the $f_+ = 5$ case without the inclusion of ionic aggregates, shown in the middle row of Fig. S2, we can see again the most probable solvation structure is 3EC+2EMC, with 2EC+2EMC being the next most likely. Overall, the error distribution of the solvation structures is more uniform, but the errors correspond to over 100% relative to MD simulations. This a failure of the theory to accurately predict the concentrations has its origins in neglecting the ionic aggregates (because the concentration of free cations is not correctly described), but overall the solvation distribution is qualitatively better.

Finally, we show the example of the sticky-cation theory with $f_+ = 4$ (ignoring ionic associations), as seen in the bottom row of Fig. S2. In this case, the most probably solvation environment is 2EC+2EMC and the distribution of solvation environments matches the MD simulations reasonably well, apart from the fact that only 4-coordinated structures are predicted. Similar to the previous case, the errors are $\sim$100% because of the neglected ionic associations not predicting the concentration of free cations accurately.

Overall, the full $f_+ = 6$ case is the most quantitative, even if the solvation distribution is not qualitatively the best. The other cases of the theory might better capture the trends in the solvation environments, but they are quantitatively not as accurate.

In Fig. S3 we show the solvation distribution for ion pairs, $c_{11sq}$, from MD and theory, in a) and b), respectively. In the theory $f_+ = 6$ is used, as this appears to match the data better when ionic associations are accounted for. We find that the theory and MD match reasonably well, with the most probable solvation environment being 2EC+2EMC. In Fig. S3 we also show the ionic aggregate distribution. This has been calculated through

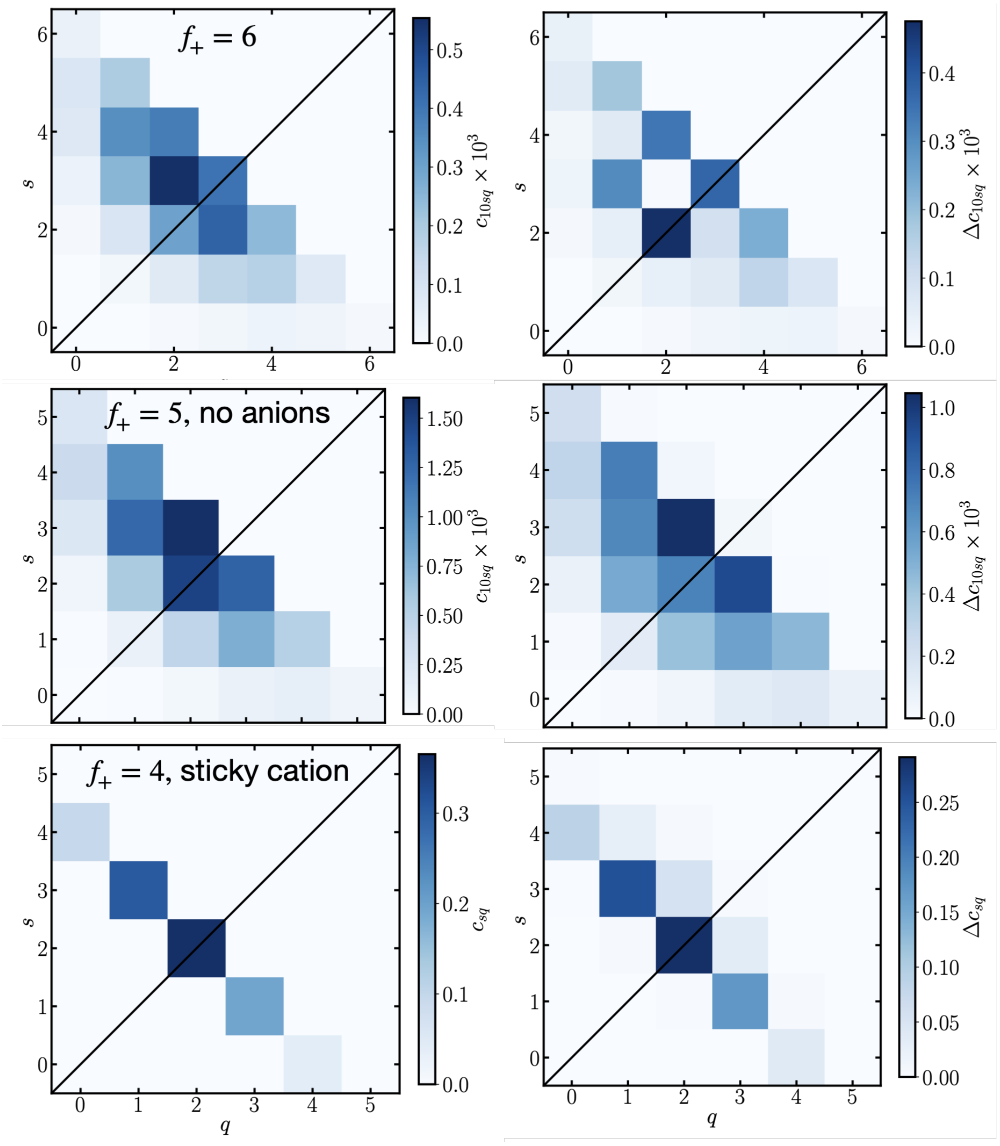


FIG. S2. Solvation distributions for $Li^+$ cations in the bulk for various approximations of the theory (left), with the absolute difference with the MD solvation distribution shown (right).

$$c_{lm} = \sum_{sq} c_{lmsq}, \tag{S45}$$

such that the solvation distributions are summed-out, leaving on the ionic backbone information. We find that ion pairs exist in appreciable quantities, but larger aggregates are only present in small concentrations. The theory underestimates the number of ion pairs and overpredicts slightly the larger aggregates.

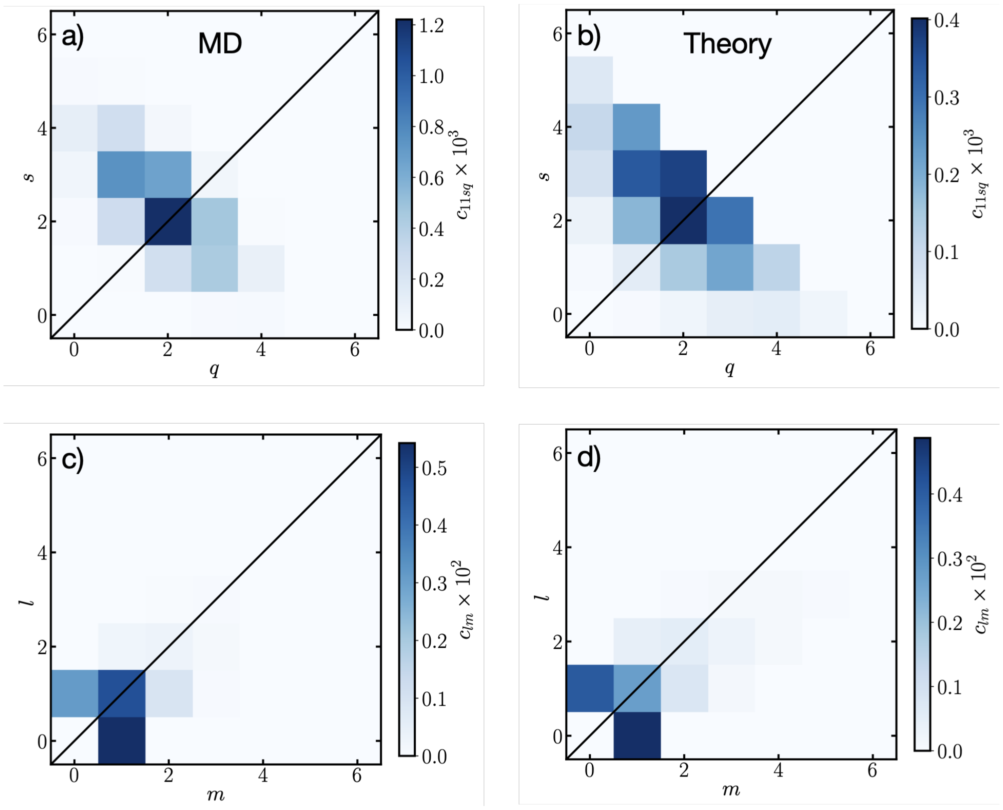


FIG. S3. Solvation distributions for ion pairs, $c_{11sq}$, in the bulk from MD a) and theory b) as a function of the number of coordinating EC ($s$) and EMC ($q$). Ionic aggregate distribution, $c_{lm}$, as a function of the number of cations $l$ and anions $m$ from bulk MD c) and theory d).

### *2. Diffuse*

In Fig. S4 we show the the absolute difference between the MD simulation and theory predicted solvation distribution for the diffuse EDL. In the theory, we use the full case with $f_+ = 5$, and discussed the solvation distributions in the main text. Overall, the agreement with the MD simulations is better than the bulk, with errors reaching 50% for worst-case. We systematically observe the theory underpredict the concentration of the 3EC+2EMC environment.

In Fig. S5 we show the ion pair solvation environments in the diffuse EDL of negative surfaces. At -0.4 $e\text{nm}^{-2}$, the MD simulations prefer EC in the solvation shell, with 3EC, 3EC+EMC and 2EC+EMC also being common solvation environments. The theory predicts the 3EC+EMC environment to be the most likely. For -0.6 $e\text{nm}^{-2}$ very few ion pairs exist, but -0.8 $e\text{nm}^{-2}$ has more than the -0.4 $e\text{nm}^{-2}$ case. The MD simulations and theory agree

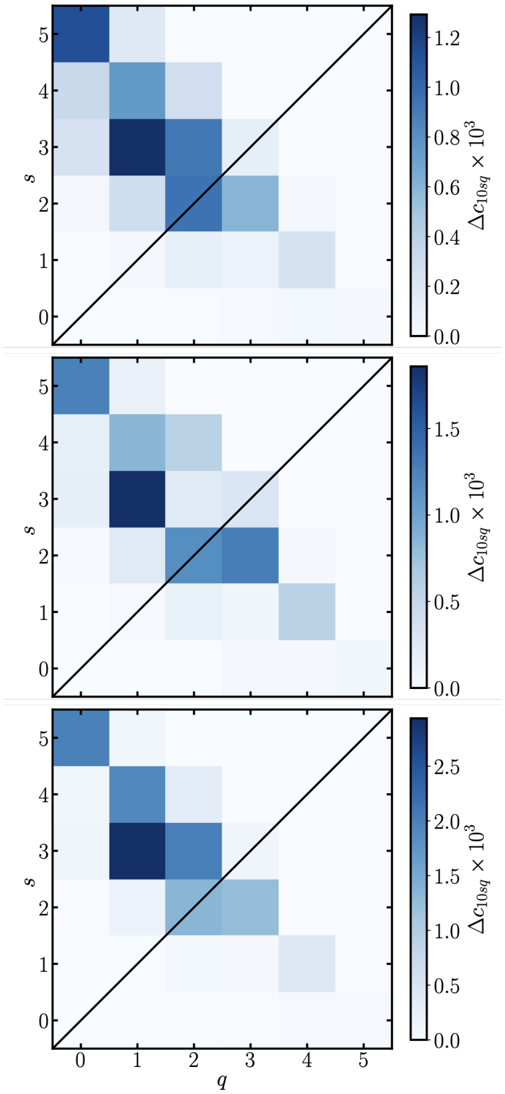


FIG. S4. Difference between theory and MD simulations for solvation distributions of $Li^+$ in the diffuse EDL, with the top, middle and bottom rows corresponding to -0.4 $e\mathrm{nm}^{-2}$, -0.6 $e\mathrm{nm}^{-2}$ and -0.8 $e\mathrm{nm}^{-2}$, respectively.

on the most likely solvation environments for the -0.8 $e\mathrm{nm}^{-2}$ case, although the predicted concentrations are quite different.

In Fig. S6, we show the ionic aggregate distributions in the diffuse EDL of negative electrodes. Overall, fewer ion pairs and aggregates are present in the diffuse EDL of negative electrodes.

In Fig. S7, we show the solvation distribution of $Li^+$ in the diffuse EDL of positive

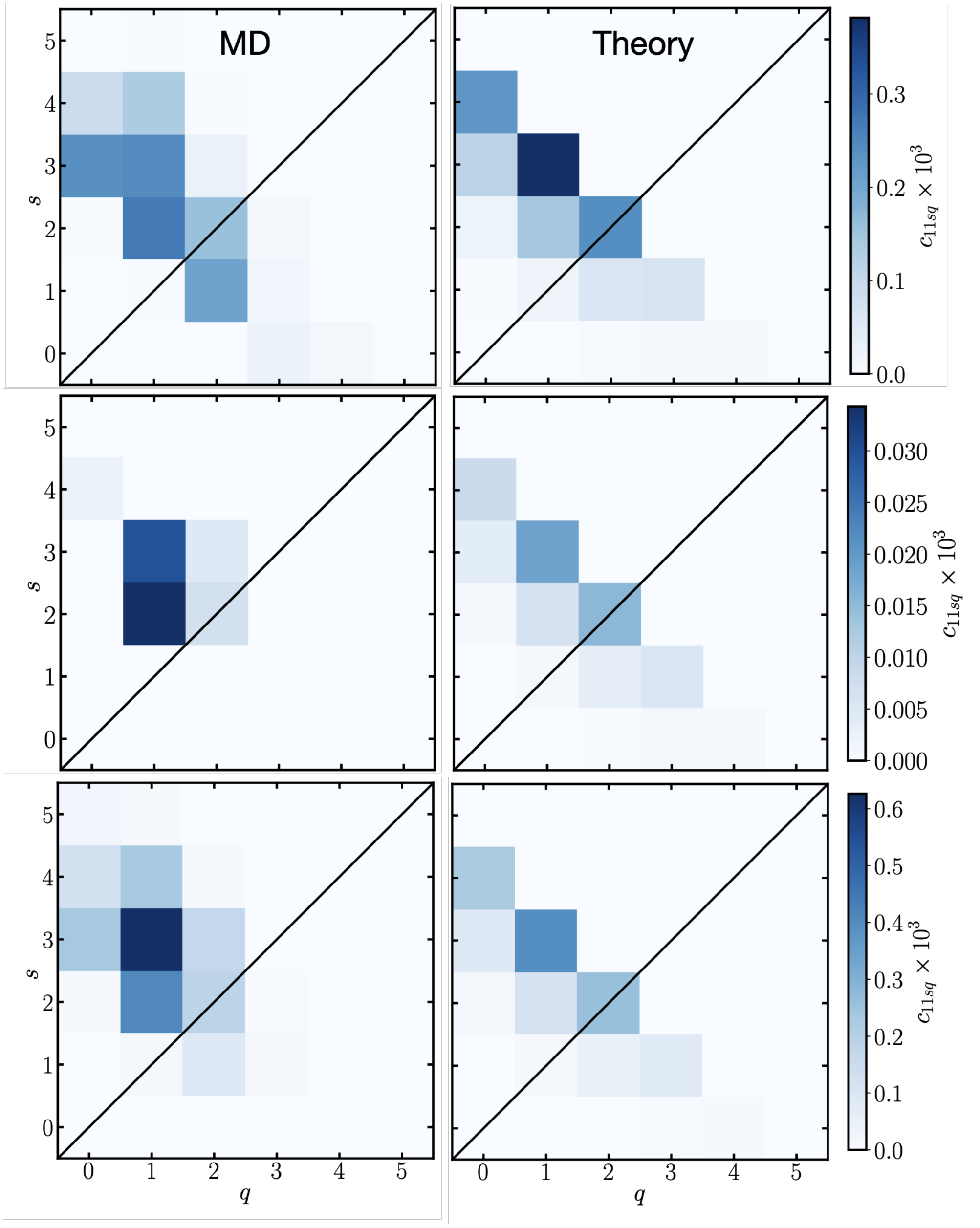


FIG. S5. Solvation distributions for ion pairs, $c_{11sq}$, in the diffuse EDL from MD and theory, as a function of the number of coordinating EC ($s$) and EMC ($q$), with the top, middle and bottom rows corresponding to -0.4 $e$nm$^{-2}$, -0.6 $e$nm$^{-2}$ and -0.8 $e$nm$^{-2}$, respectively.

electrodes. As to be expected, overall the concentrations of these clusters are lower than that of the negative electrode. For 0.4 $e$nm$^{-2}$, the MD simulation predicts 2EC+2EMC to be the most common environment, whereas the theory predicts 2EC+3EMC. For the more positive surface charges, the solvation distributions shift more towards EMC. The theory can capture this trend, but as we are using $f_+ = 5$, similar to the negative diffuse EDL, we

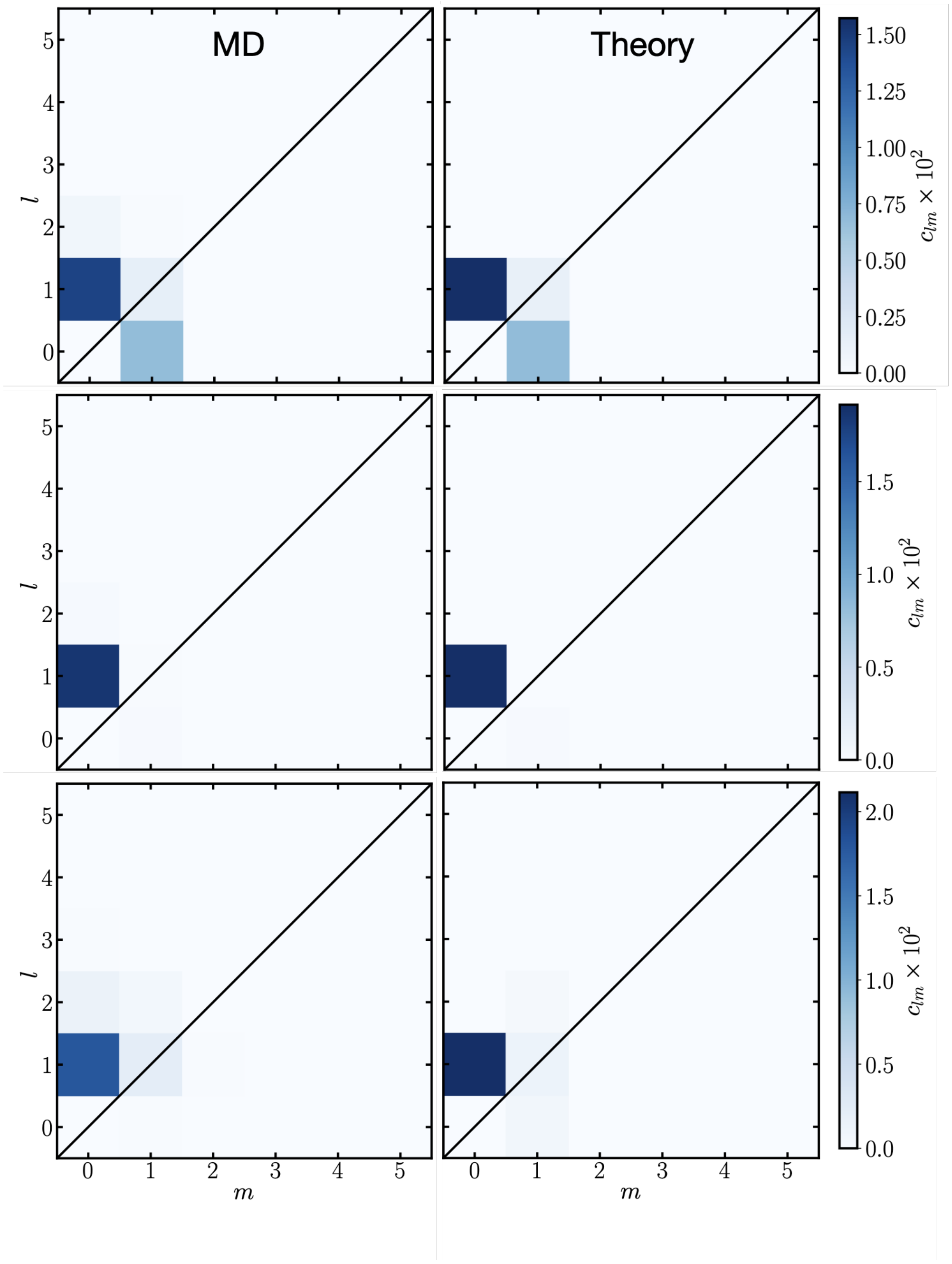


FIG. S6. Ionic aggregate distributions in the diffuse EDL from MD and theory, with the top, middle and bottom rows corresponding to -0.4 $e$nm$^{-2}$, -0.6 $e$nm$^{-2}$ and -0.8 $e$nm$^{-2}$, respectively.

are predicting larger coordination shells than the MD simulations.

In Fig. S8, we show the solvation environments of the ion pairs in the diffuse EDL of positive electrodes. The concentrations of these species is larger than that of negative electrodes. For 0.4 $e$nm$^{-2}$, the MD simulations predict 2EC+2EMC and EC+3EMC to be

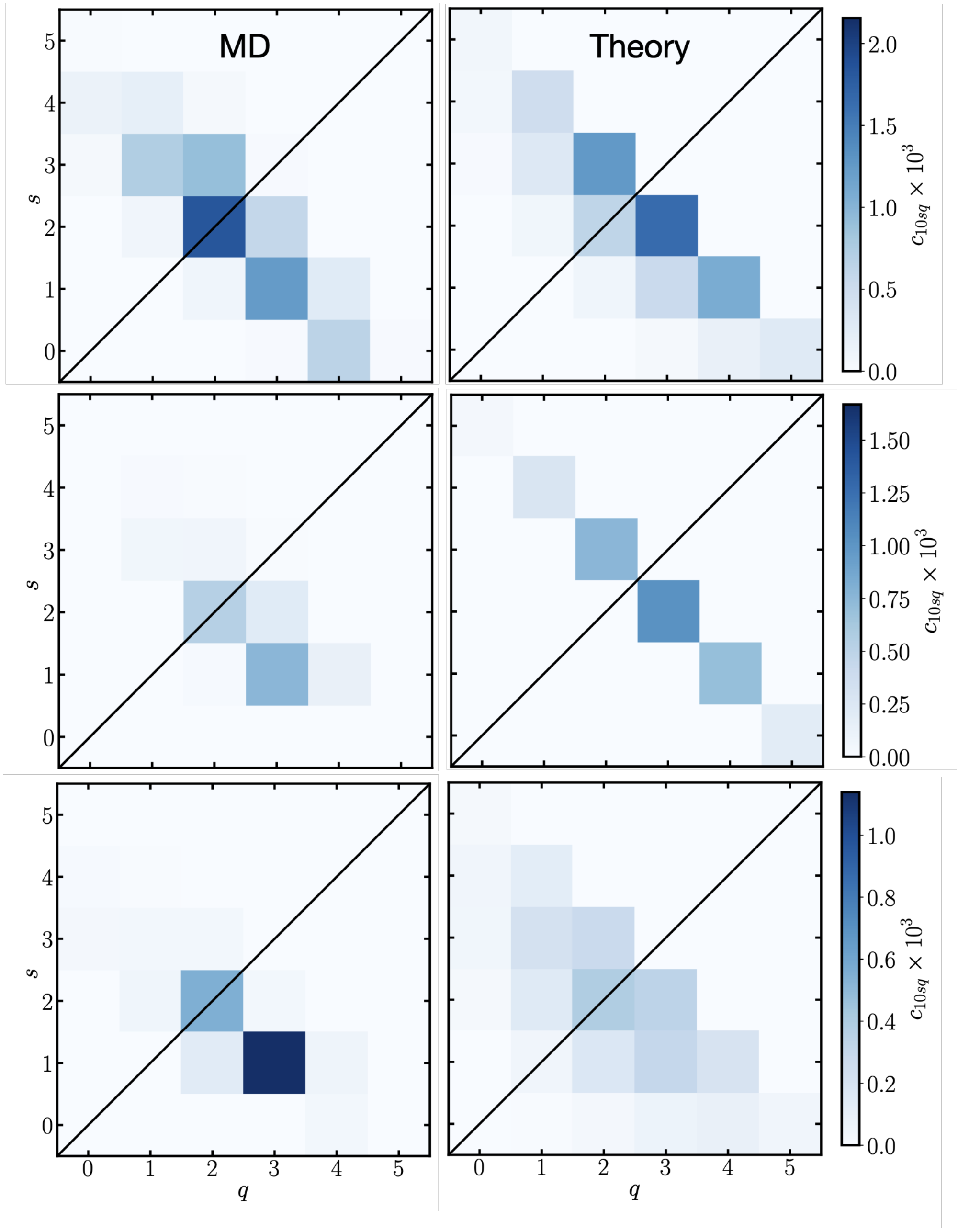


FIG. S7. Solvation distributions of $Li^+$, $c_{10sq}$, in the positive diffuse EDL from MD and theory, as a function of the number of coordinating EC ($s$) and EMC ($q$), with the top, middle and bottom rows corresponding to 0.4 $e$nm$^{-2}$, 0.6 $e$nm$^{-2}$ and 0.8 $e$nm$^{-2}$, respectively.

the most likely environments, and the theory is able to reproduce this, albeit with lower concentrations. A similar observation holds for 0.6 $e$nm$^{-2}$. In the 0.8 $e$nm$^{-2}$ case, the MD simulations predict EC+2EMC to be the most likely. While the theory has a similar distribution, the concentrations are significantly lower.

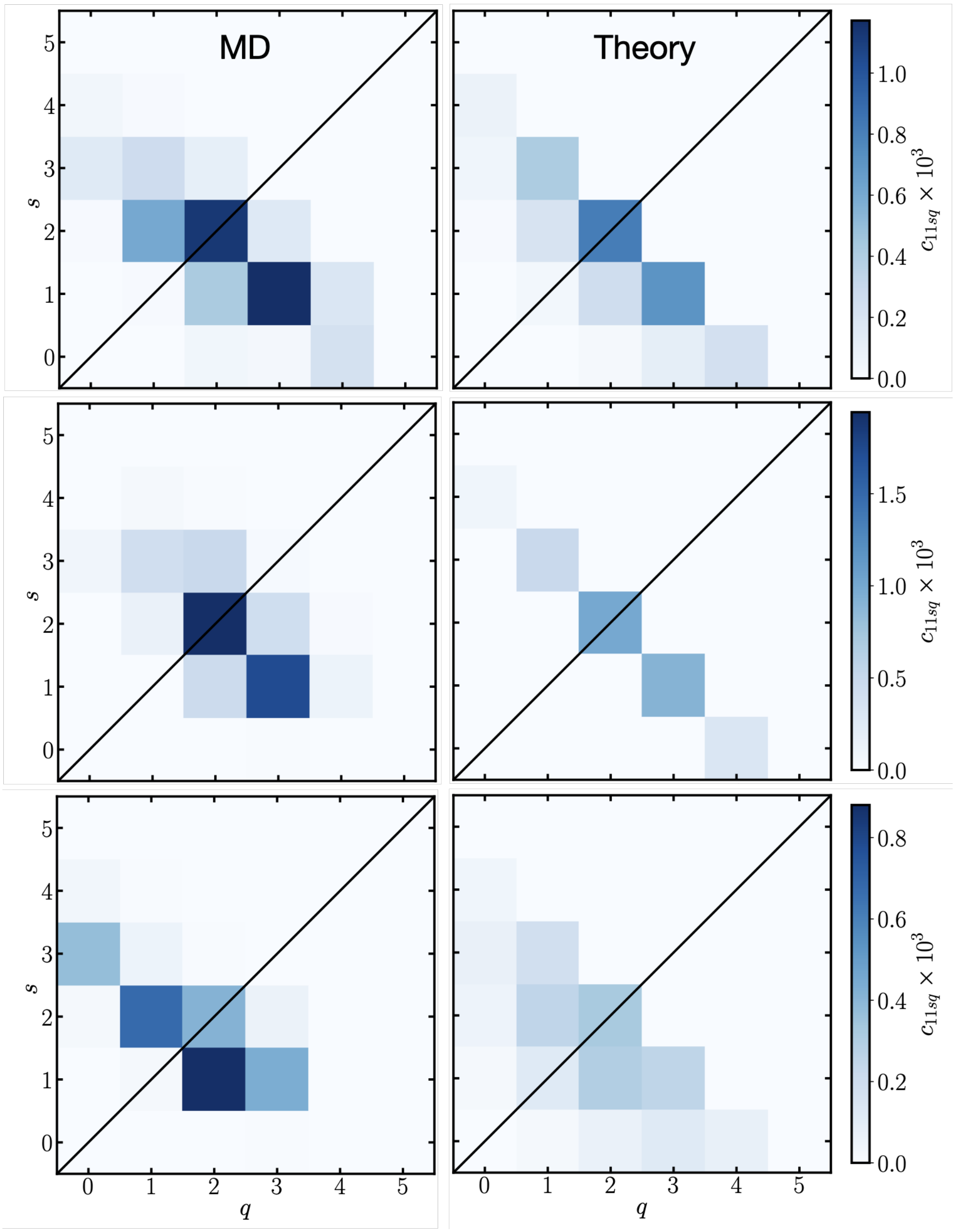


FIG. S8. Solvation distributions for ion pairs, $c_{11sq}$, in the diffuse EDL from MD and theory, as a function of the number of coordinating EC ($s$) and EMC ($q$), with the top, middle and bottom rows corresponding to 0.4 $e$nm$^{-2}$, 0.6 $e$nm$^{-2}$ and 0.8 $e$nm$^{-2}$, respectively.

In Fig. S9, we show the ionic aggregates in the positive diffuse EDL. As can be seen, there appears to be more ion pairs and larger aggregates than the negative diffuse EDL. At 0.4 $e$nm$^{-2}$, the MD simulations have significant ion pairs, and the theory reproduces these reasonably well. For 0.6 $e$nm$^{-2}$, the ionic aggregates are becoming more skewed to

negative clusters, with 3 anion clusters becoming more prominent both in MD simulations and theory. Finally, for the highest surface charge of 0.8 $e$nm$^{-2}$, free anions dominate, and there is good agreement between MD simulations and theory.

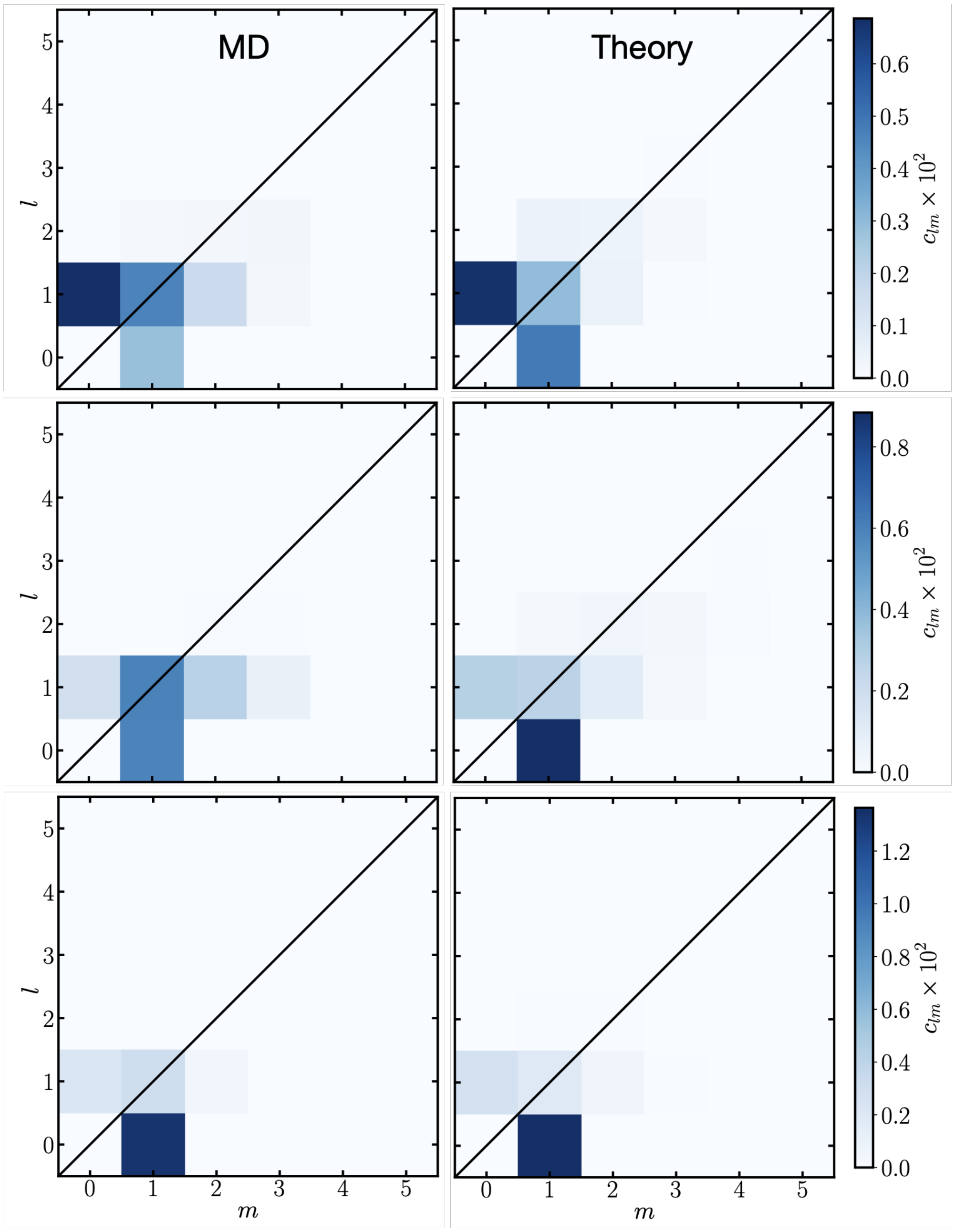


FIG. S9. Ionic aggregate distributions in the diffuse EDL from MD and theory, with the top, middle and bottom rows corresponding to 0.4 $e$nm$^{-2}$, 0.6 $e$nm$^{-2}$ and 0.8 $e$nm$^{-2}$, respectively.

### *3. Helmholtz*

In Fig. S10, we show a comparison between the solvation distribution, $c_{10sq}$, in the Helmholtz layer from MD and the (non-sticky) theory with different functionalities. We find that the most probable solvation environment is 2EC+2EMC, and both theory plots also have this. However, for $f_+ = 5$, there are significant concentrations of solvation environments with 5 and 3-2 solvents. This is not seen in the MD simulations, where most of the solvation environments contain 4 sovlents. Whereas, for the theory case of $f_+ = 4$, the distribution is significantly more peaked around 4 solvents, with not 5-solvent environments, and few 3 solvent environments. This motivates reducing the functionality in the Helmholtz layer, in addition to having association free energy which change in the Helmholtz layer.

In Fig. S11 we show the difference between MD simulations and the theory prediction. Overall, similar agreement is found to the diffuse EDL, which is better than the agreement in the bulk.

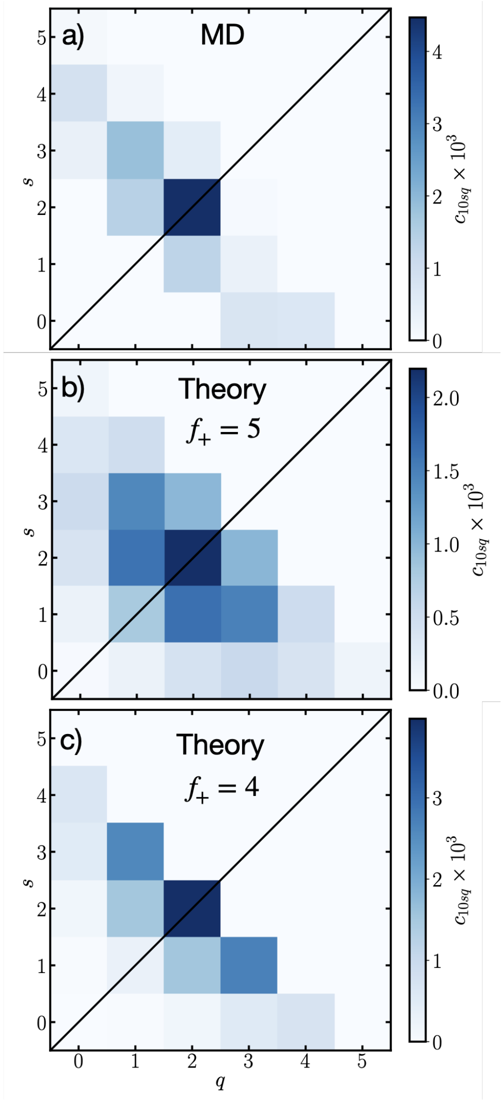


FIG. S10. Solvation distributions, $c_{10sq}$, of Li in the Helmholtz layer from MD a), with theory as a function of the number of coordinating EC ($s$) and EMC ($q$) for $f_+ = 5$ and $f_+ = 4$ in b) and c), respectively, for a surface charge of $-0.4$ enm$^{-2}$.

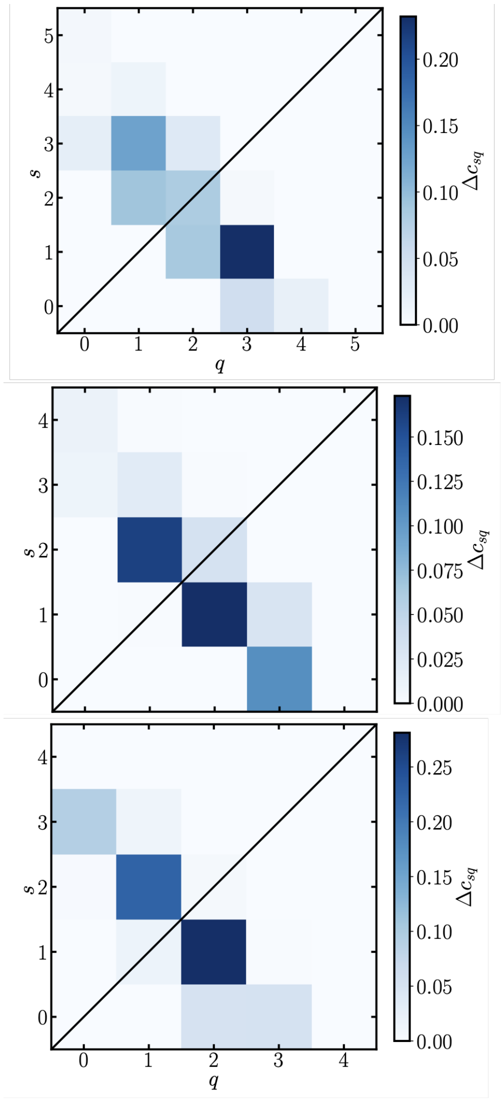


FIG. S11. Difference between theory and MD simulations for solvation distributions of $Li^+$ in the Helmholtz layer, with the top, middle and bottom rows corresponding to -0.4 $e\mathrm{nm}^{-2}$, -0.6 $e\mathrm{nm}^{-2}$ and -0.8 $e\mathrm{nm}^{-2}$, respectively.

## C. DOL+DME

### 1. Bulk

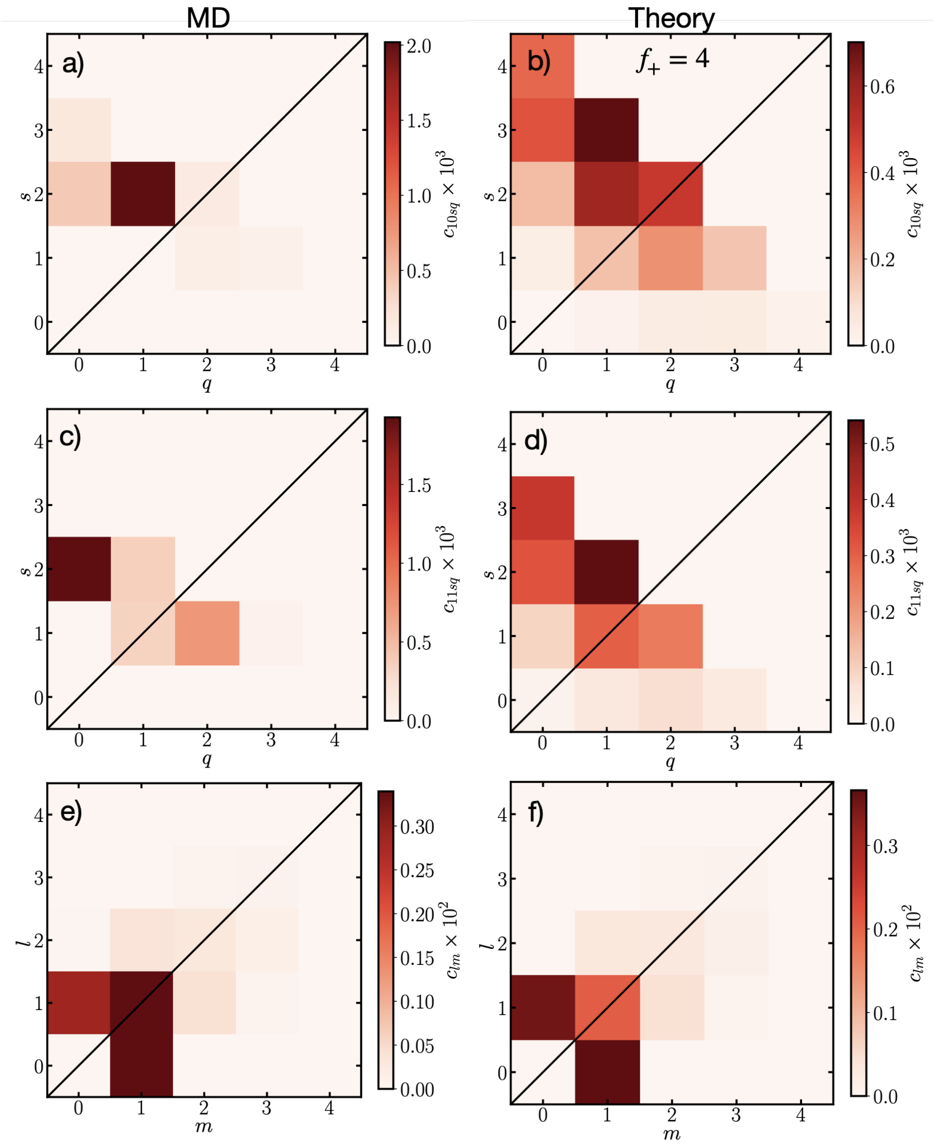


FIG. S12. Solvation distributions of Li, $c_{10sq}$, in the bulk from MD a) and theory b) as a function of the number of coordinating DME ($s$) and DOL ($q$). Solvation distributions for ion pairs, $c_{11sq}$, in the bulk from MD c) and theory d) as a function of the number of coordinating DME ($s$) and DOL ($q$). Ionic aggregate distribution, $c_{lm}$, as a function of the number of cations $l$ and anions $m$ from bulk MD e) and theory f).

In Fig. S12 we show the solvation distribution of $c_{10sq}$ from MD and theory, respectively, in a) and b). In MD we find the most probable solvation environment to be 2DME+DOL. From the MD simulations, we computed $\lambda_x = 470.4$ and $\lambda_y = 6.1$ (without ionic aggregates), which correspond to $\lambda_x/\lambda_y = 77.2$, indicating that the DME is preferred over DOL in the first coordination shell of $Li^+$, which can also be seen from $p_{+x} = 0.681$. Accounting for ionic aggregates results in $\lambda_x = 1215.5$, $\lambda_y = 15.75$, and $\lambda_x/\lambda_y = 77.2$.

In the theory, $f_+ = 4$ was used, as the average coordination number was larger than 3, and the cation-anion associations are known to be significant between Li-TFSI. The theory predicts the most probable solvation environment to be 3DME+DOL, with 2DME+DOL the next most likely. If the solvent environments are just considered, $f_+ = 3$ might fit the data better. In c) and d), we show the solvation environments for ion pairs, $c_{11sq}$, from MD and theory. The most probable environment in MD is now 2DME, while the theory predicts 2DME+DOL. In Fig. S12 we also show the ionic aggregate distribution, $c_{lm}$. This has been calculated through

$$c_{lm} = \sum_{sq} c_{lmsq}, \tag{S46}$$

such that the solvation distributions are summed-out, leaving on the ionic backbone information. We find that ion pairs exist in appreciable quantities in MD, but larger aggregates do not. The theory predicts fewer ion pairs than observed in MD, which is a consequence of the concentration of ion pairs having to be smaller than the concentration of free ions.

### 2. *Diffuse*

In Fig. S13 we show the solvation distributions of $Li^+$ in the diffuse EDL for various surface charges from MD and theory. Similar to the bulk case, the match between theory and MD is reasonable, but using $f_+ = 4$ does not yield the most accurate match with MD. In Tab. S1, we display the computed association constants, probability and mole ratios. As can be seen, there is a slight reduction of $\lambda_x/\lambda_y$ with more negative surfaces charges, and the $p_{+x}$ is also remaining approximately constant.

In Fig. S14 we show the ionic aggregate distribution, $c_{lm}$, at various surface positive charges. At $+0.4$ $e$nm$^{-2}$, we find only few free cations, with free anions and ion pairs dominating the distribution, and some $c_{12}$ aggregates. For the intermediate surface charge of

$+0.4$ $e$nm$^{-2}$, we find free anions dominate, with significant concentrations of $c_{11}$ and $c_{21}$, and also free cations. Finally, at the largest surface charge of $+0.8$ $e$nm$^{-2}$, free anions dominate with only few ion pairs and free cations, with practically no larger aggregates. This non-monotonic change in the aggregate distribution suggests electric-field induced associations are occurring, which is further discussed in the main text. The theory is able to reproduce some of the observed trends, but it appears to struggle with the ion pair concentration most.

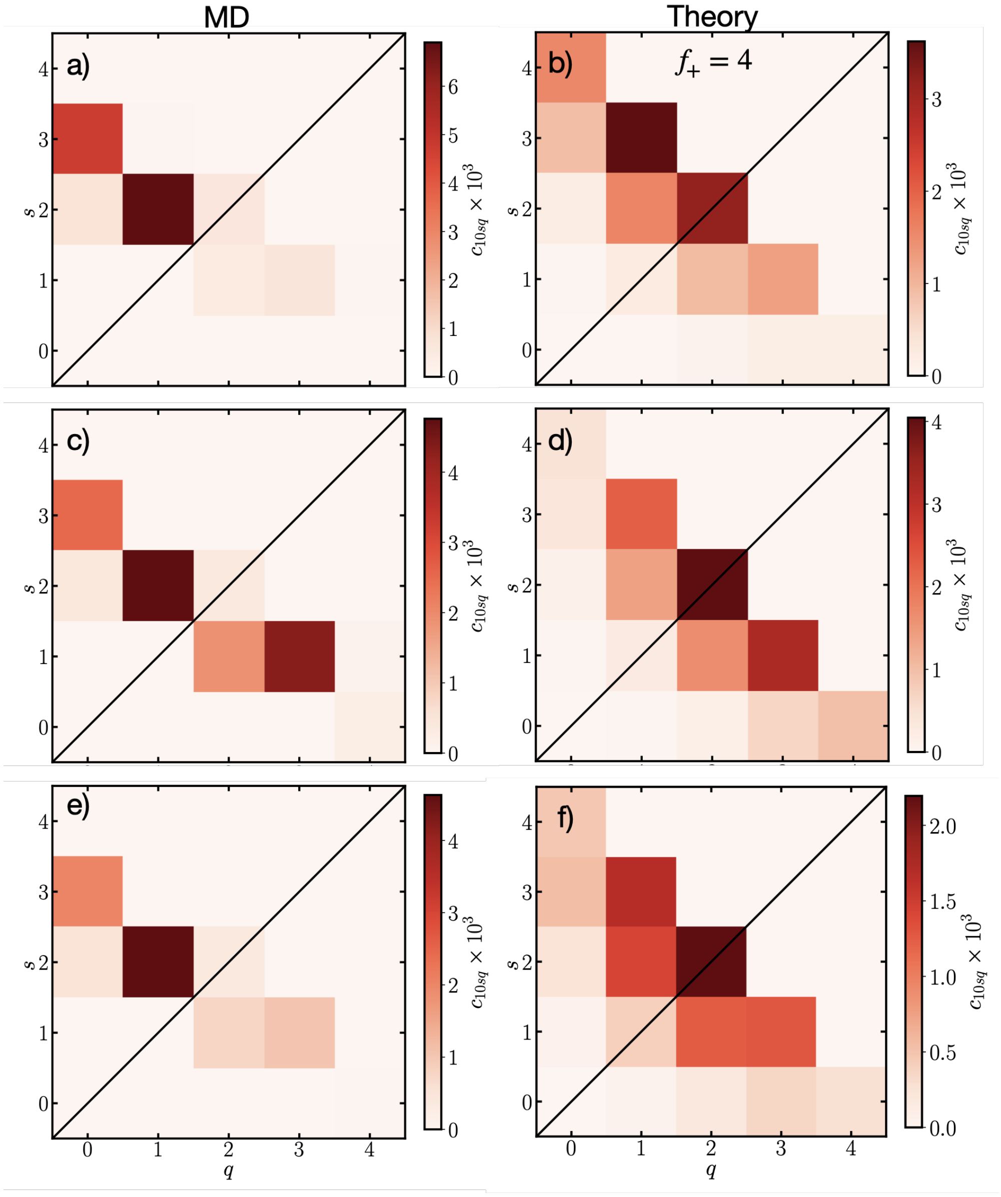


FIG. S13. Solvation distributions of Li, $c_{10sq}$, in the diffuse EDL from MD [a),c),e)] and theory [b),d),f)] as a function of the number of coordinating DME ($s$) and DOL ($q$) at, respectively, surface charges of $-0.4$, $-0.6$ and $-0.8$ $e$nm$^{-2}$.

| $\sigma/$ $e$nm$^{-2}$ | $\lambda_x/\lambda_y$ | $x_x/x_y$ | $p_{+x}$ |
|---|---|---|---|
| -0.4 | 87.25 | 0.731 | 0.650 |
| -0.6 | 10.50 | 0.378 | 0.517 |
| -0.8 | 19.76 | 0.318 | 0.554 |

TABLE S1. Summary of association constant ratios and mole fraction ratios for the DME and DOL solvents for the diffuse EDL at the indicated surface charges.

### 3. *Helmholtz*

In Fig. S15 we show the solvation distribution in the Helmholtz layer for various surface charges, from MD and theory. The MD simulations most probable solvation environment at $-0.4$ $e$nm$^{-2}$ is 2DME+DOL, which is the same as the bulk. At $-0.6$ $e$m$^{-2}$, the distribution becomes significantly more skewed to DOL solvation environments, with 3DOL and 3DOL+DME being the most probable, with DME+DOL and 2DME+DOL being the next most likely. Finally, at $-0.8$ $e$nm$^{-2}$ DME+DOL and 3DOL are the most probable environments. This is in contrast to the EC-EMC case, where the solvation distributions remained approximately constant. Similar to the EC-EMC case, however, there is a clear trend to reducing numbers of solvents in the shell of $Li^+$ with increasing negative surface charges, with 3 and 2 solvents becoming more probable.

In Tab. S2 we report the computed association constants, mole ratios of the solvent and association probability in the Helmholtz layer of DME+DOL. As can be seen, $\lambda_x/\lambda_y$ is strongly reduced in comparison to the bulk, and also smaller than the diffuse EDL. Moreover, there is a strong reduction of $p_{+x}$, indicating there must be some breakdown of the assumptions of the simplified model outlined in the main text. We don't find that there is a strong preference for either solvent in the Helmholtz layer.

For the theory case, we choose $f_+ = 3$. For $-0.4$ $e$nm$^{-2}$ the agreement is good between theory and MD, which highlights the fact that a functionality of 3 might work better in the bulk. For $-0.6$ and $-0.8$ $e$nm$^{-2}$, the agreement is less good, although the general shapes of the distributions are captured.

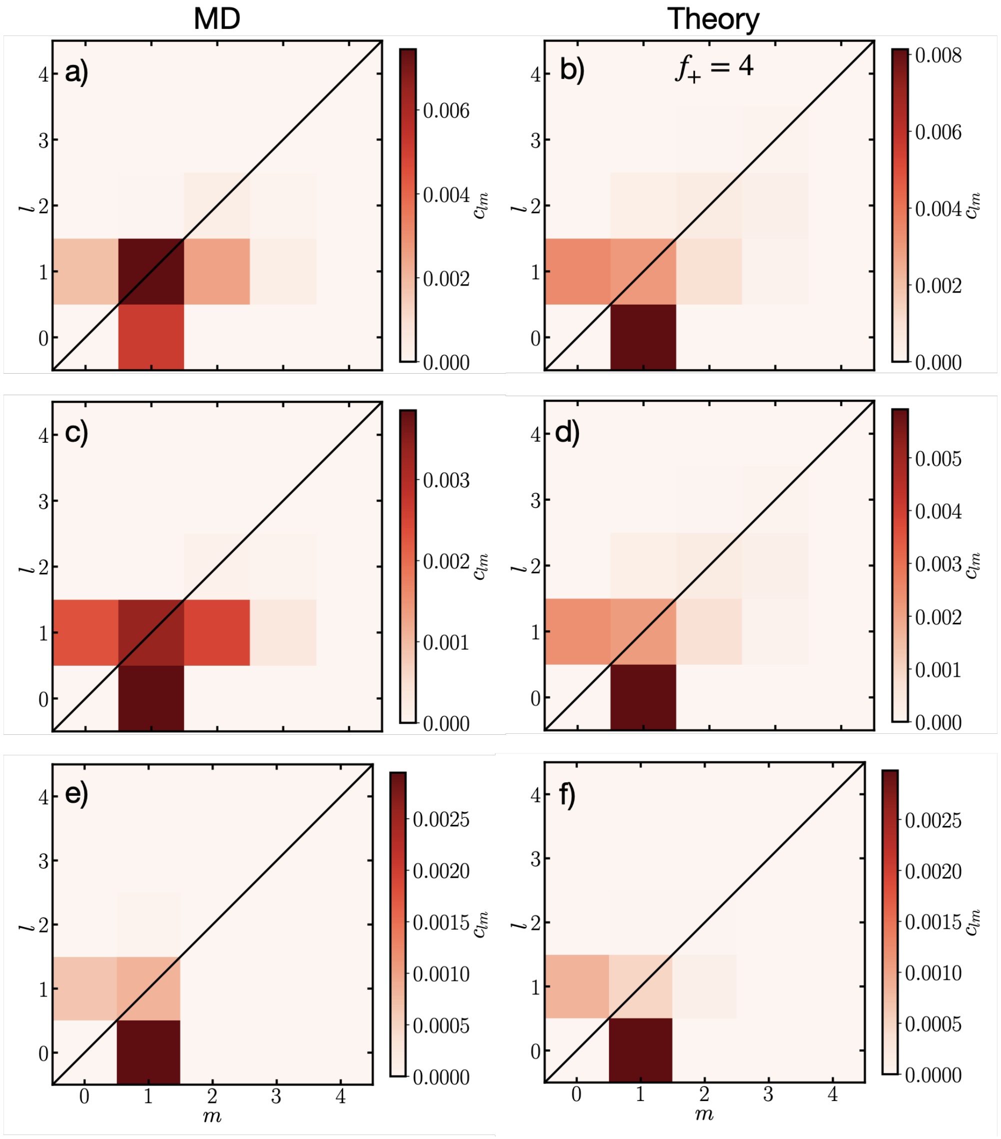


FIG. S14. Ionic aggregate distribution, $c_{lm}$, in the diffuse EDL from MD [a),c),e)] and theory [b),d),f)] as a function of the number cations ($l$) and anions ($m$) at, respectively, surface charges of +0.4, +0.6 and +0.8 $e$nm$^{-2}$.

| $\sigma$/ $e$nm$^{-2}$ | $\lambda_x/\lambda_y$ | $x_x/x_y$ | $p_{+x}$ |
|---|---|---|---|
| -0.4 | 5.895 | 0.384 | 0.603 |
| -0.6 | 2.425 | 0.258 | 0.320 |
| -0.8 | 1.340 | 0.256 | 0.197 |

TABLE S2. Summary of association constant ratios and mole fraction ratios for the DME and DOL solvents for the Helmholtz layer at the indicated surface charges.

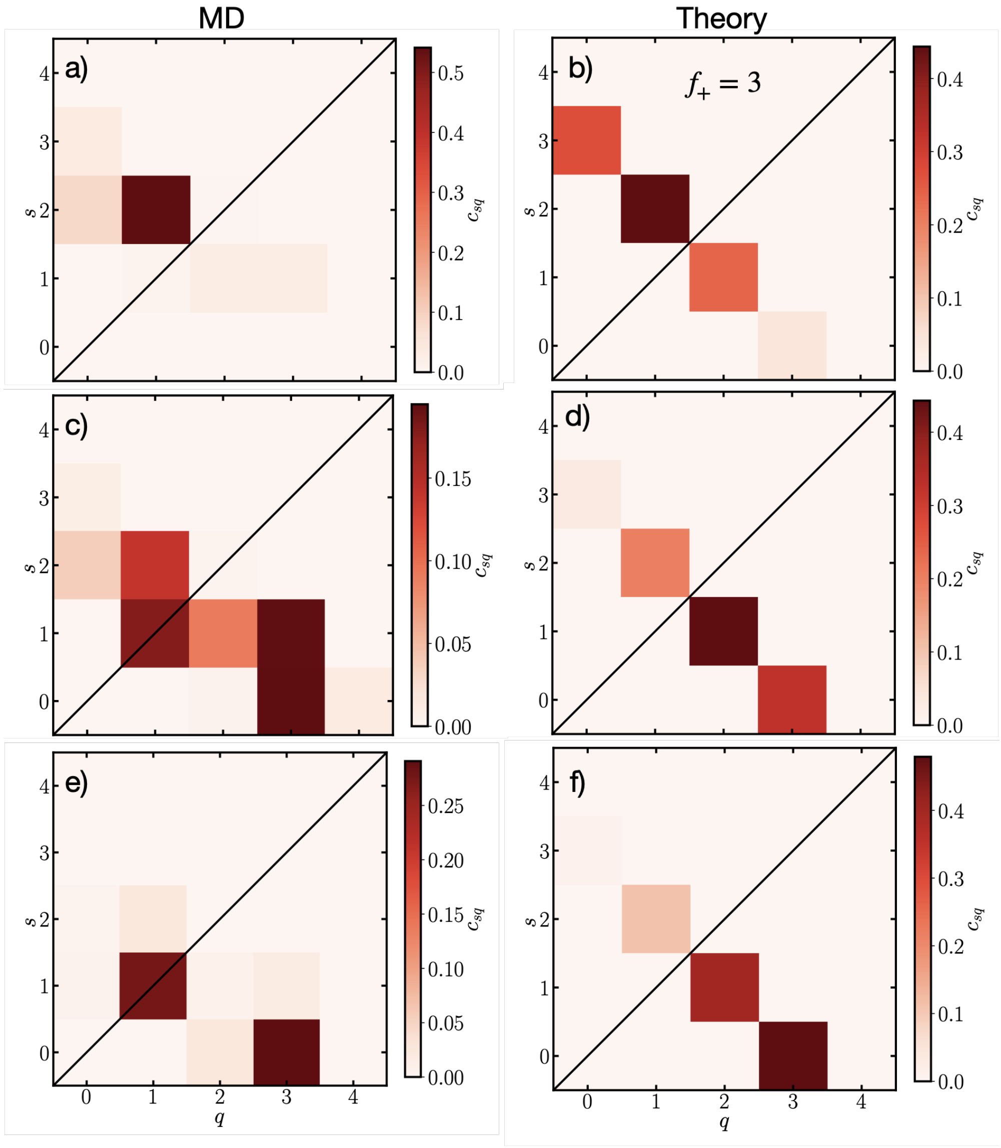


FIG. S15. Solvation distribution of Li, $c_{10sq}$, in the Helmholtz layer from MD [a),c),e)] and Theory [b),d),f)] as a function of the number of coordinating EC ($s$) and EMC ($q$) at, respectively, surface charges of $-0.4$, $-0.6$ and $-0.8$ enm$^{-2}$.

## D. EC+EMC+FEC

### 1. *Bulk*

In Fig. S16 we show the solvent distributions of Li, $c_{10sqp}$, for various FEC solvent coordination ($p$). Overall, the results are similar to the main text, with $f_+ = 5$ in the theory matching the MD simulations well. We computed the association constants to be $\lambda_x = 132.6$, $\lambda_y = 46.8$ and $\lambda_z = 143.0$, not accounting for ionic aggregation effects, only solvation, which is what was done in the main text for this solvent. We find $\lambda_x/\lambda_y = 2.8$, which is approximately the value in the absence of FEC, and $\lambda_x/\lambda_z = 0.9$ which would be expected from the fact that EC and FEC have similar solvation properties. In addition, we find $p_{+x} = 0.456$, $p_{+y} = 0.408$ and $p_{+z} = 0.135$ in the sticky approximation, where the large difference in $p_{+x}$ and $p_{+z}$ comes from the large difference in the amounts of each solvent present.

### 2. *Diffuse*

Similar to the bulk case, the diffuse EDl solvation distributions from MD match those from theory well, as seen in Figs. S17-S19. In Tab. S3 we report the computed parameters for the theory, where we find similar behavior to the main text for the diffuse EDL.

| $\sigma$/ enm$^{-2}$ | $\lambda_x/\lambda_y$ | $\lambda_x/\lambda_z$ | $x_x/x_y$ | $x_x/x_z$ | $p_{+x}$ | $p_{+y}$ |
|---|---|---|---|---|---|---|
| -0.4 | 4.171 | 1.628 | 0.624 | 3.190 | 0.488 | 0.385 |
| -0.6 | 4.317 | 0.727 | 0.799 | 4.014 | 0.538 | 0.314 |
| -0.8 | 6.704 | 2.287 | 0.890 | 3.197 | 0.541 | 0.319 |

TABLE S3. Summary of association constant ratios and mole fraction ratios for the EC, EMC and FEC solvents for the diffuse EDL at the indicated surface charges.

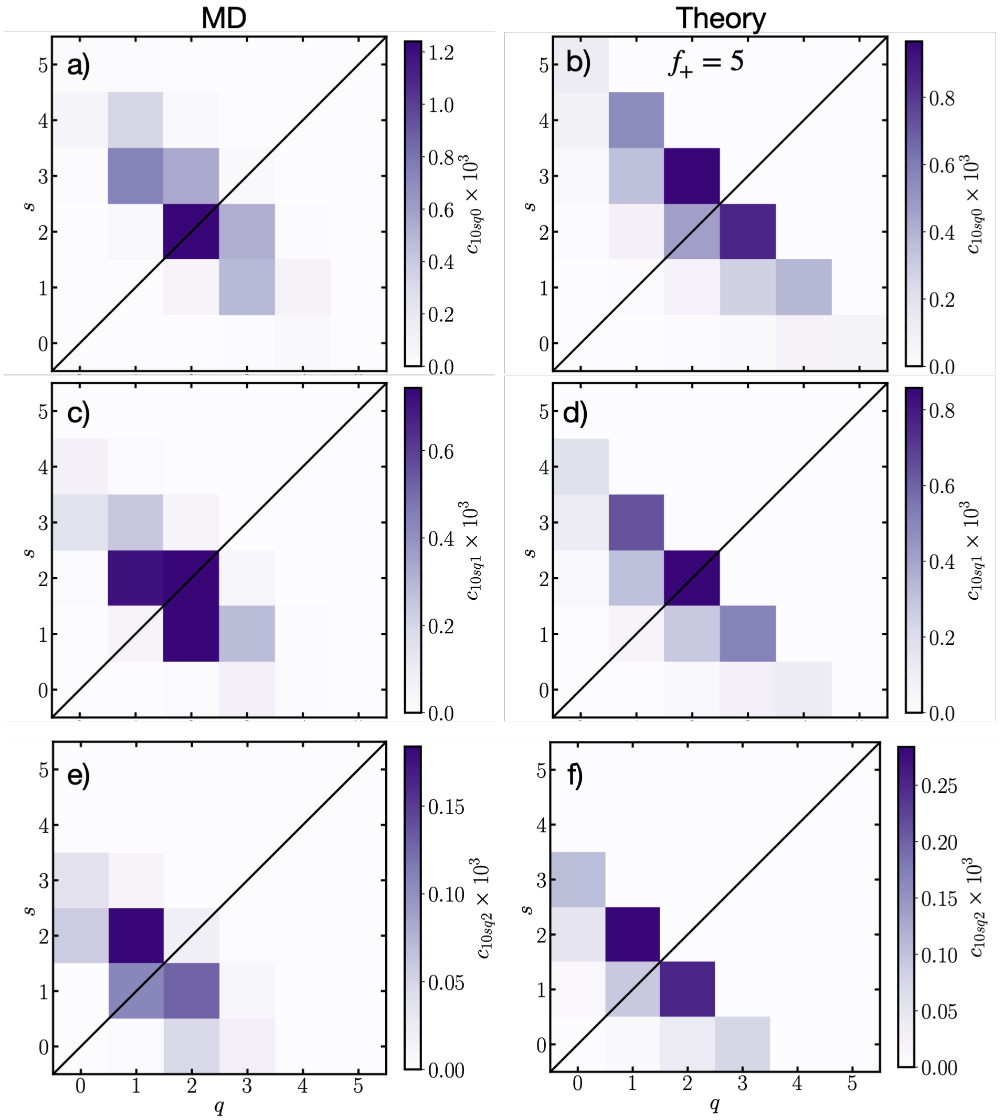


FIG. S16. Solvation distribution of Li, $c_{10sqp}$, in the bulk from MD [a),c),e)] and Theory [b),d),f)] as a function of the number of coordinating EC ($s$) and EMC ($q$) with, respectively, 0, 1 and 2 coordinating FEC solvents ($p$).

### *3. Helmholtz*

Again to the bulk and diffuse EDL cases, the Helmholtz layer solvation distributions from MD match those from theory well, as seen in Figs. S20-S22. Similarly, in Tab. S4 we report the parameters for the theory in the Helmholtz layer. Again, we find $\lambda_x/\lambda_y$ strongly decreases with more negative surfaces charges, with $\lambda_x/\lambda_z$ remaining approximately constant. We find $\lambda_x/\lambda_z$ increases, although not as much as in the main text, as $x_x/x_z$ is also increasing. While

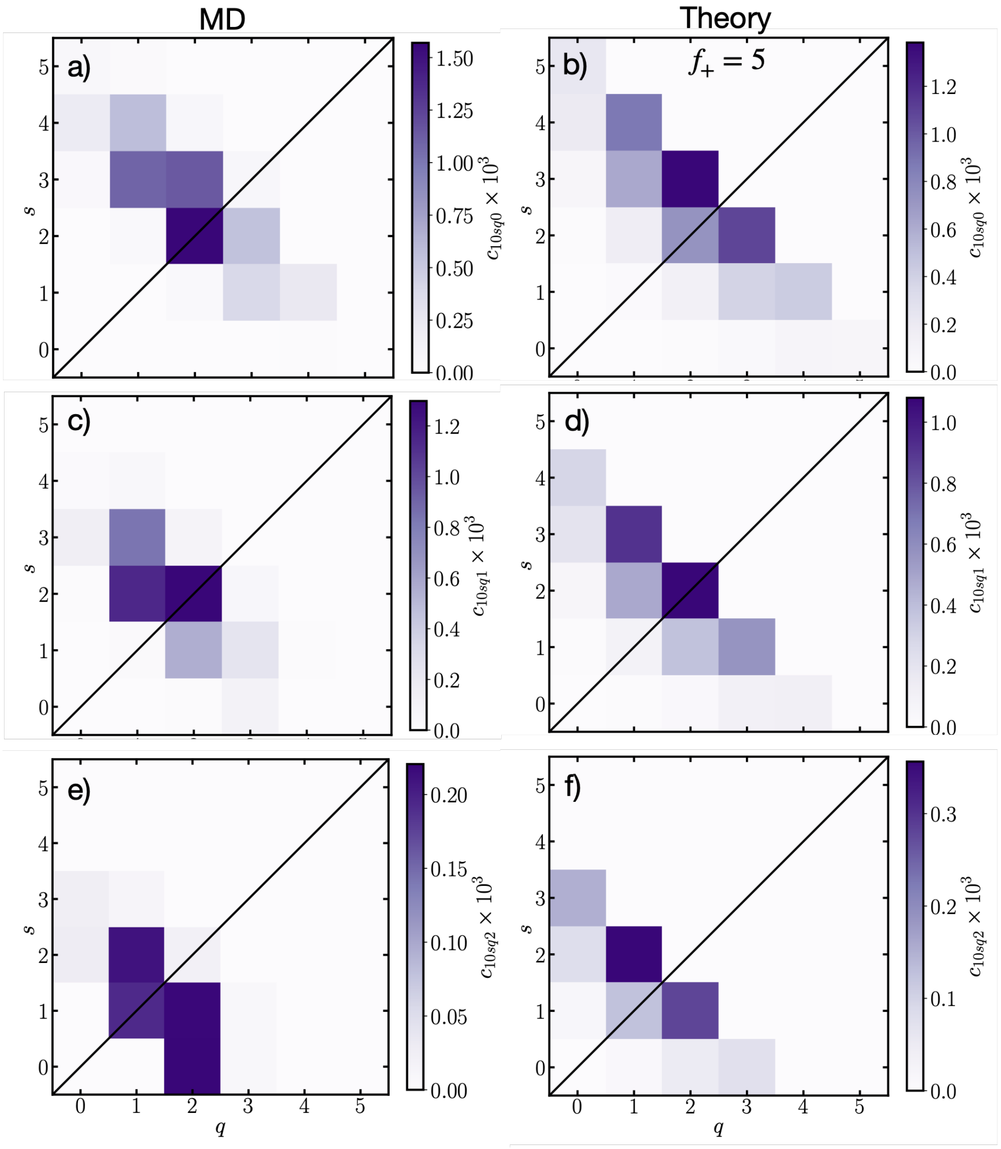


FIG. S17. Solvation distribution of Li, $c_{10sqp}$, in the diffuse layer from MD at $-0.4$ $e$nm$^{-2}$ [a),c),e)] and Theory [b),d),f)] as a function of the number of coordinating EC ($s$) and EMC ($q$) with, respectively, 0, 1 and 2 coordinating FEC solvents ($p$).

the association probabilities are not as constant as the 2-solvent case in the main text, they are still reasonably constant, suggesting the assumptions of the simplified theory in the main text are at least approximately holding.

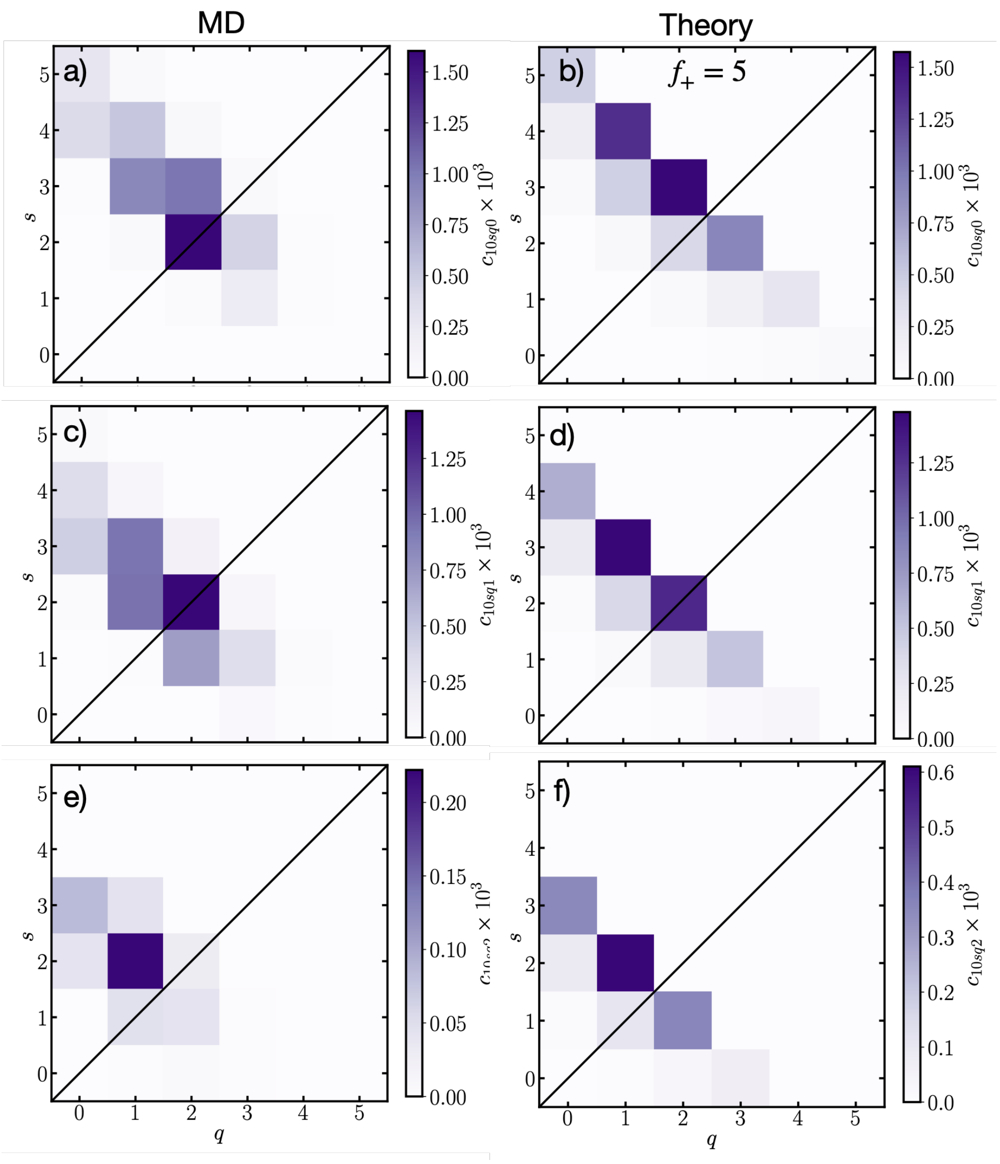


FIG. S18. Solvation distribution of Li, $c_{10sqp}$, in the diffuse layer from MD at $-0.6\ e\mathrm{nm}^{-2}$ [a),c),e)] and Theory [b),d),f)] as a function of the number of coordinating EC ($s$) and EMC ($q$) with, respectively, 0, 1 and 2 coordinating FEC solvents ($p$).

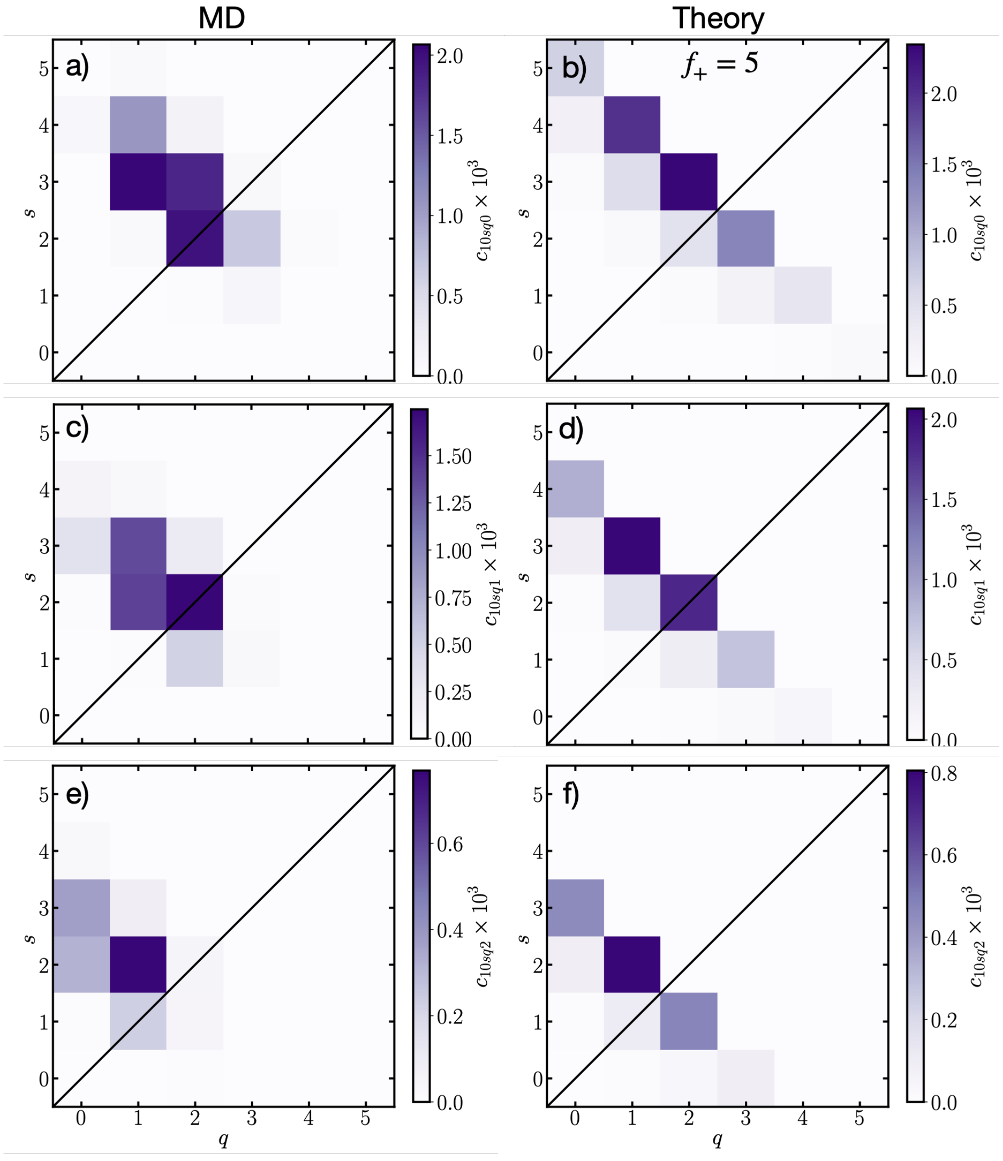


FIG. S19. Solvation distribution of Li, $c_{10sqp}$, in the diffuse layer from MD at $-0.8$ $e$nm$^{-2}$ [a),c),e)] and Theory [b),d),f)] as a function of the number of coordinating EC ($s$) and EMC ($q$) with, respectively, 0, 1 and 2 coordinating FEC solvents ($p$).

| $\sigma$/ $e$nm$^{-2}$ | $\lambda_x/\lambda_y$ | $\lambda_x/\lambda_z$ | $x_x/x_y$ | $x_x/x_z$ | $p_{+x}$ | $p_{+y}$ |
|---|---|---|---|---|---|---|
| -0.4 | 0.400 | 0.760 | 1.534 | 2.078 | 0.402 | 0.377 |
| -0.6 | 0.120 | 0.504 | 1.481 | 2.058 | 0.316 | 0.464 |
| -0.8 | 0.001 | 0.530 | 1.889 | 3.793 | 0.269 | 0.618 |

TABLE S4. Summary of association constant ratios and mole fraction ratios for the EC, EMC and FEC solvents for the Helmholtz layer at the indicated surface charges.

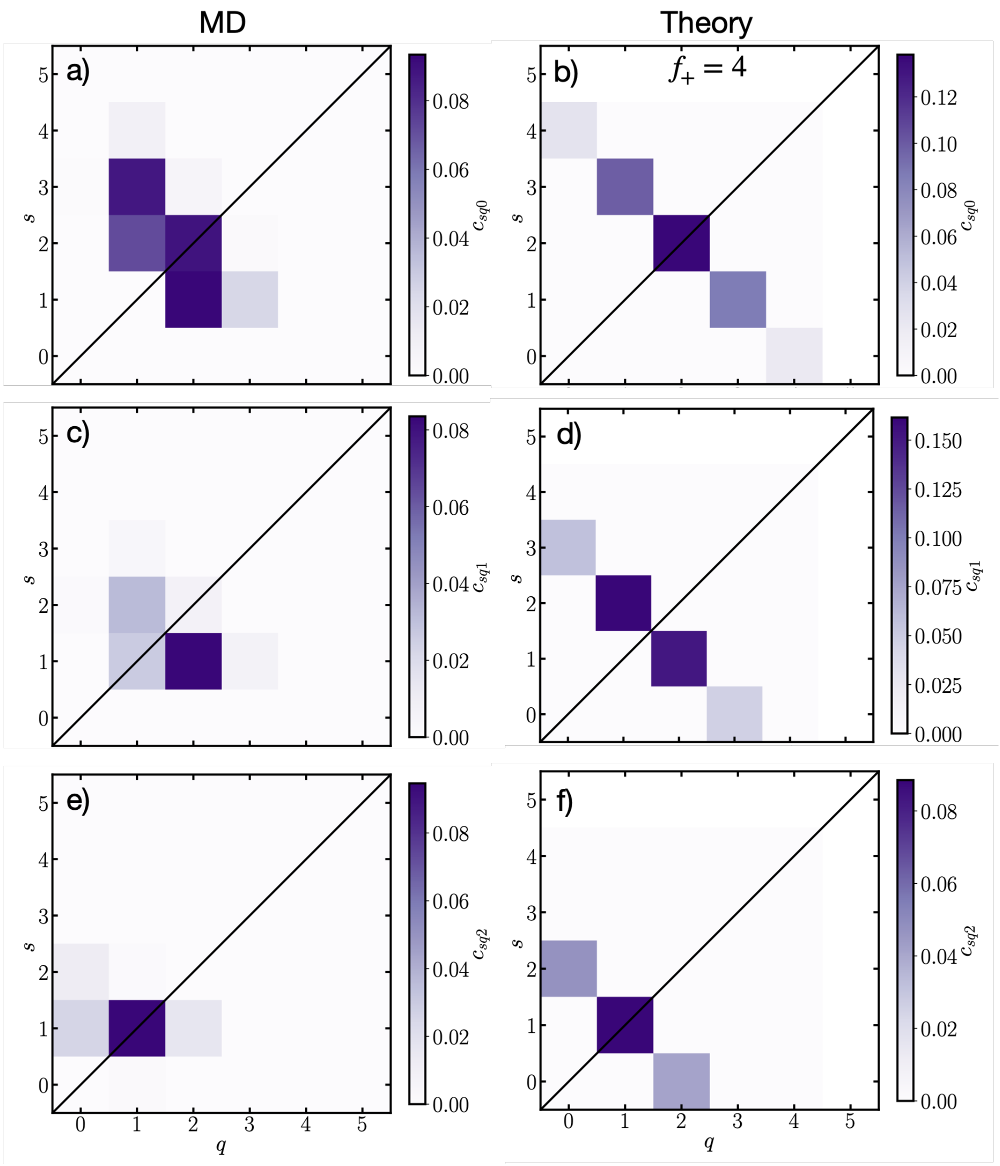


FIG. S20. Solvation distribution of Li, $c_{sqp}$, in the Helmholtz layer from MD at $-0.4$ $e\text{nm}^{-2}$ [a),c),e)] and Theory [b),d),f)] in the sticky-cation approximation as a function of the number of coordinating EC ($s$) and EMC ($q$) with, respectively, 0, 1 and 2 coordinating FEC solvents ($p$).

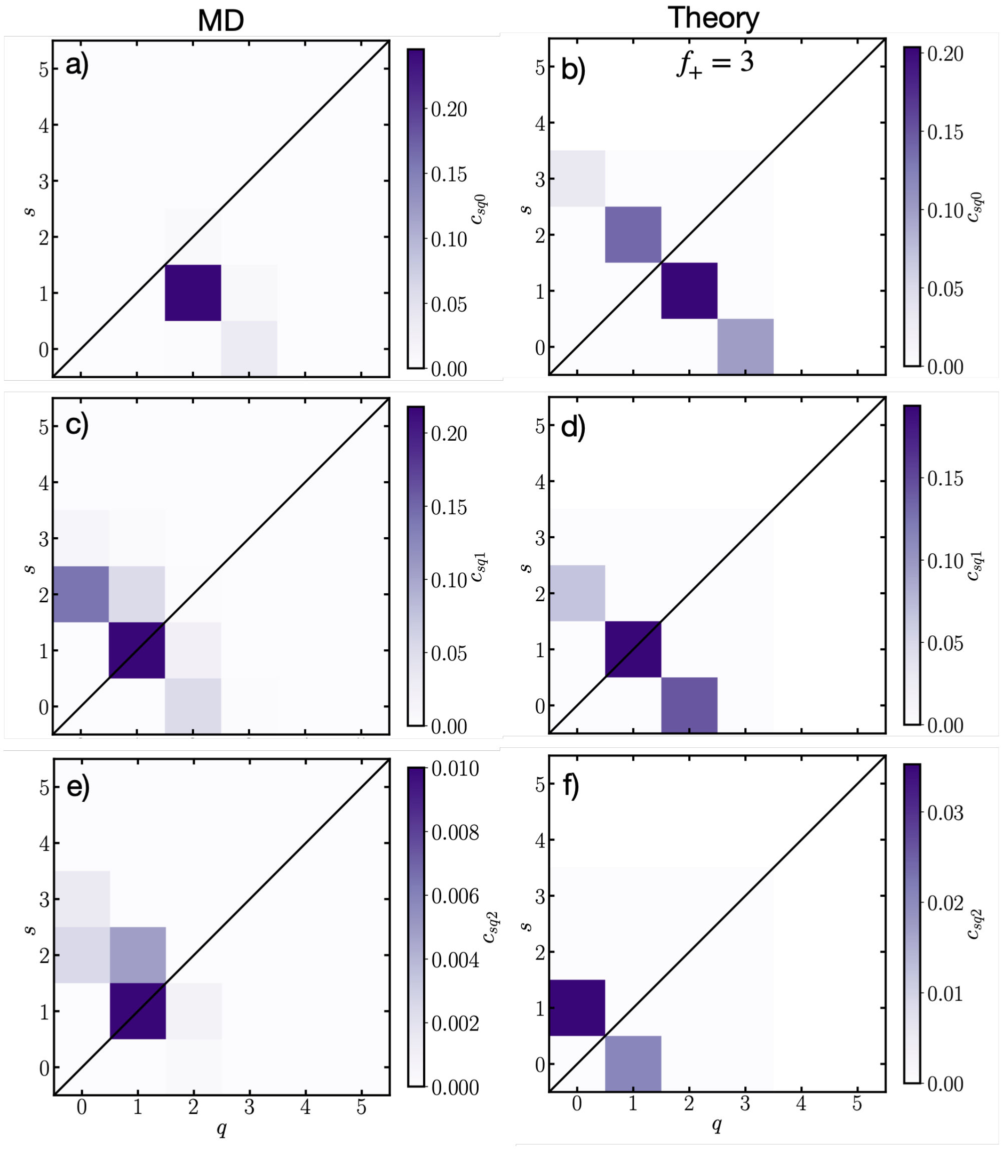


FIG. S21. Solvation distribution of Li, $c_{sqp}$, in the Helmholtz layer from MD at $-0.6$ $e$nm$^{-2}$ [a),c),e)] and Theory [b),d),f)] in the sticky-cation approximation as a function of the number of coordinating EC ($s$) and EMC ($q$) with, respectively, 0, 1 and 2 coordinating FEC solvents ($p$).

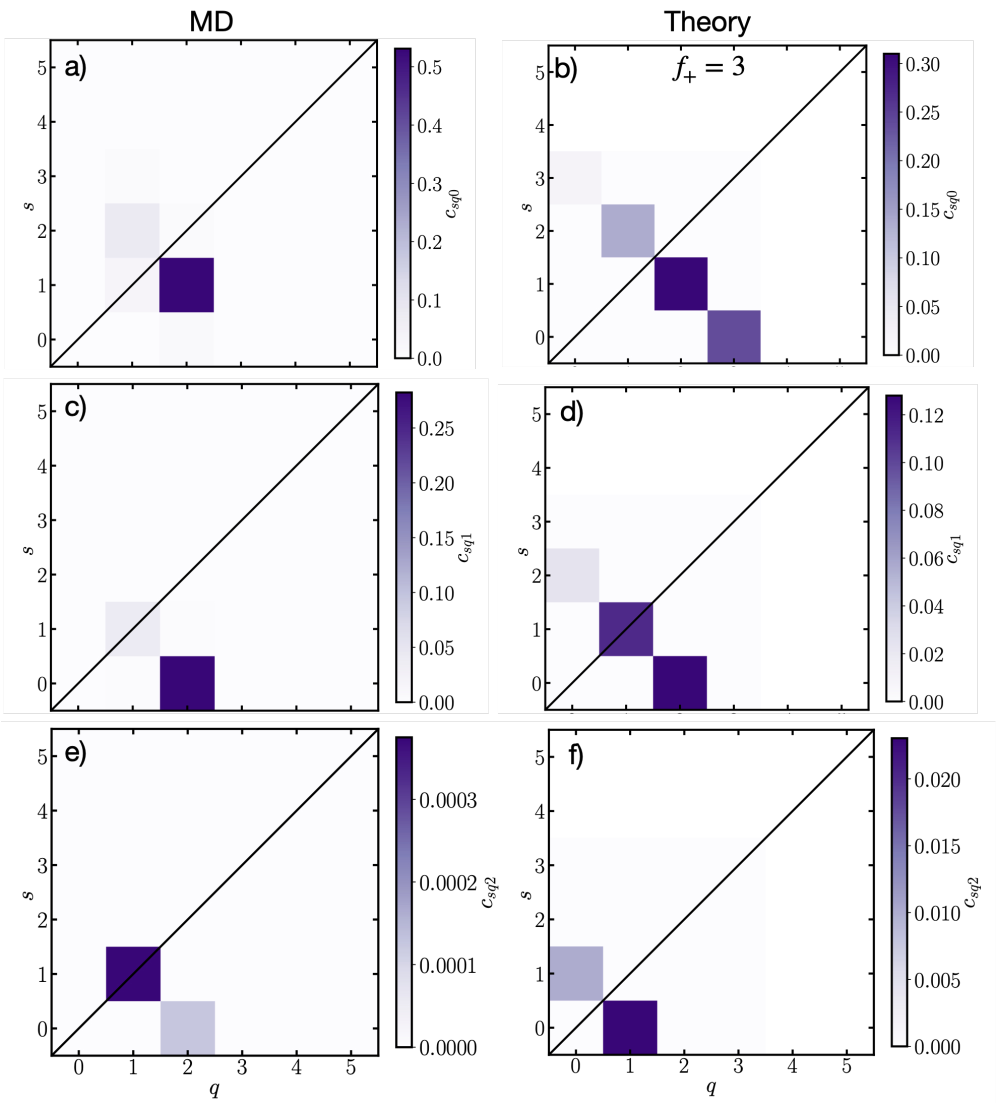


FIG. S22. Solvation distribution of Li, $c_{sqp}$, in the Helmholtz layer from MD at $-0.8\ e\text{nm}^{-2}$ [a),c),e)] and Theory [b),d),f)] in the sticky-cation approximation as a function of the number of coordinating EC ($s$) and EMC ($q$) with, respectively, 0, 1 and 2 coordinating FEC solvents ($p$).

## E. DOL+DME+FEC

### 1. *Bulk*

In Fig. S23 we show the solvation distributions for the DME-DOL-FEC case in the bulk, from MD and theory. Similar to the DME-DOL case, a functionality of $f_+ = 4$ does not match the MD simulation well for $p = 0$, with $p = 1$ also not agreeing well (albeit slightly better than the $p = 0$ case), but for $p = 2$ the agreement is better. Here we computed the association constants to be $\lambda_x = 122.2$, $\lambda_y = 3.8$ and $\lambda_z = 134.7$. We find a similar ratio of $\lambda_x/\lambda_y$ to the bulk case without FEC. Moreover, we found $p_{+x} = 0.501$, $p_{+y} = 0.227$ and $p_{+z} = 0.272$ in the sticky approximation.

### 2. *Diffuse*

Similar to the bulk case, mixed agreement between MD and theory is found for the diffuse layer, as seen in Figs. S24-S26. Analogous behaviour for the bulk and diffuse EDL has consistently been found. In Tab. S5 we present the parameters computed from MD for the theory. Overall, we find a slight reduction in $\lambda_x/\lambda_y$, but $\lambda_x/\lambda_z$ is relatively constant, and the association probabilities remaining relatively constant.

| $\sigma$/ enm$^{-2}$ | $\lambda_x/\lambda_y$ | $\lambda_x/\lambda_z$ | $x_x/x_y$ | $x_x/x_z$ | $p_{+x}$ | $p_{+y}$ |
|---|---|---|---|---|---|---|
| -0.4 | 12.975 | 0.721 | 0.404 | 2.863 | 0.480 | 0.342 |
| -0.6 | 9.612 | 0.305 | 0.331 | 1.700 | 0.383 | 0.336 |
| -0.8 | 11.643 | 0.323 | 0.230 | 6.241 | 0.506 | 0.377 |

TABLE S5. Summary of association constant ratios and mole fraction ratios for the DME, DOL and FEC solvents for the diffuse EDL at the indicated surface charges.

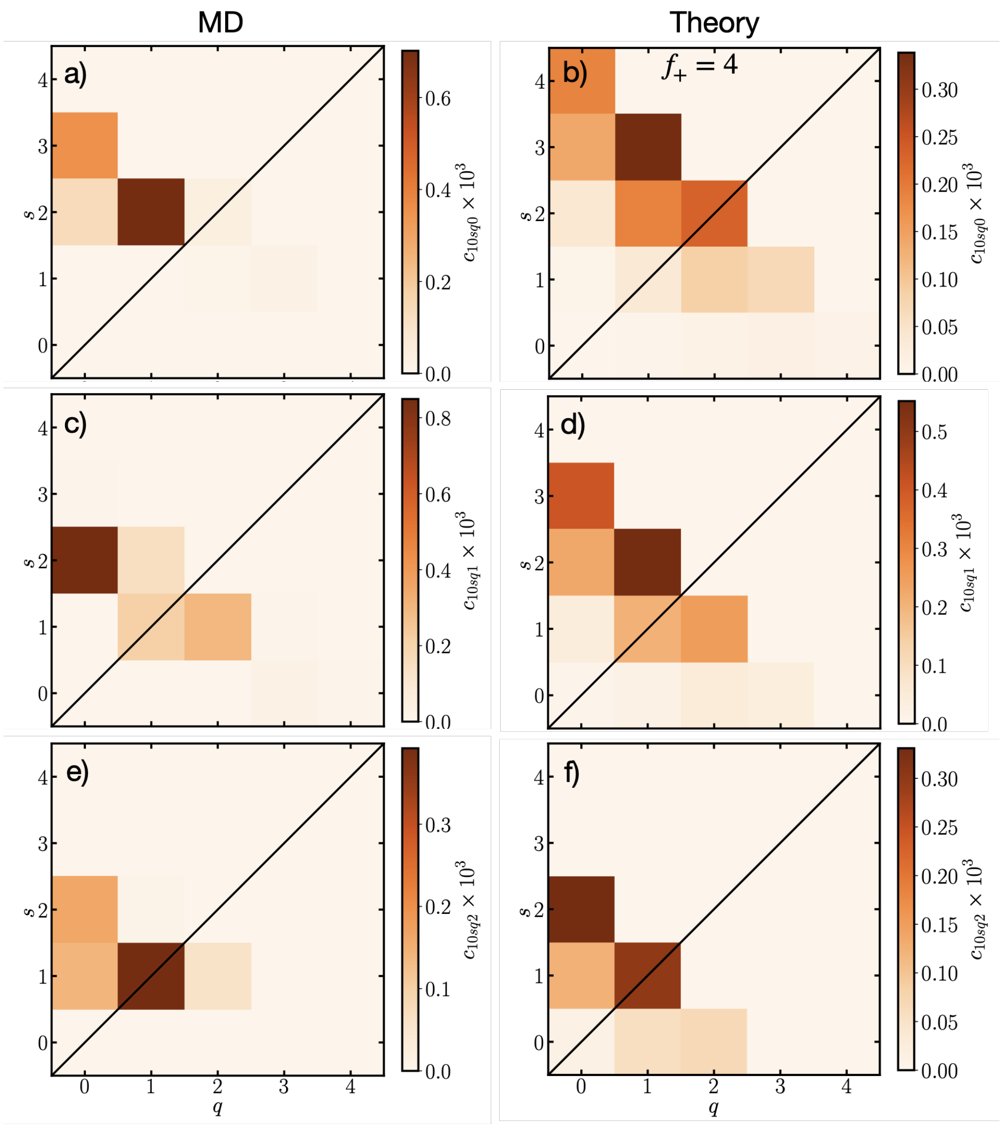


FIG. S23. Solvation distribution of Li, $c_{10sqp}$, in the bulk from MD [a),c),e)] and theory [b),d),f)] as a function of the number of coordinating DME ($s$) and DOL ($q$) with, respectively, 0, 1 and 2 coordinating FEC solvents ($p$).

In Fig. S27 we show the ionic aggregate distribution for the DME-DOL-FEC case, where we find analogous results to the DME-DOL case in the diffuse EDL.

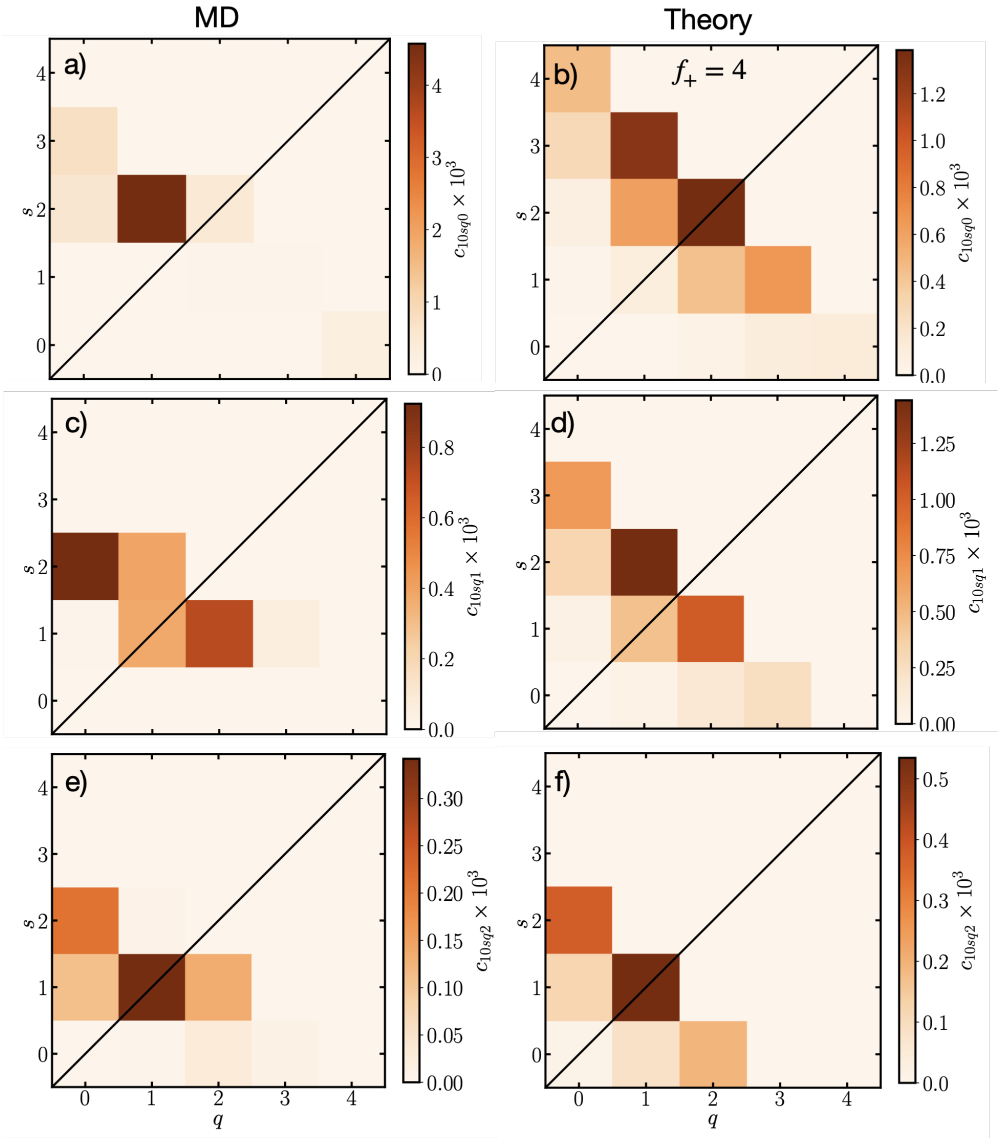


FIG. S24. Solvation distribution of Li, $c_{10sqp}$, in the diffuse layer from MD at $-0.4$ $e$nm$^{-2}$ [a),c),e)] and Theory [b),d),f)] as a function of the number of coordinating DME ($s$) and DOL ($q$) with, respectively, 0, 1 and 2 coordinating FEC solvents ($p$).

### *3. Helmholtz*

In Fig. S28 we show the solvent distributions in the Helmholtz layer at $-0.4$ $e$nm$^{-2}$ for DME-DOL-FEC. In the theory we use $f_+ = 3$, which again might match the bulk solvent distributions equally well. Overall, our agreement between theory and MD is reasonable, with the main discrepancy being in $c_{sq2}$, where MD finds more solvents coordinated, although the concentration of these cases is very small.

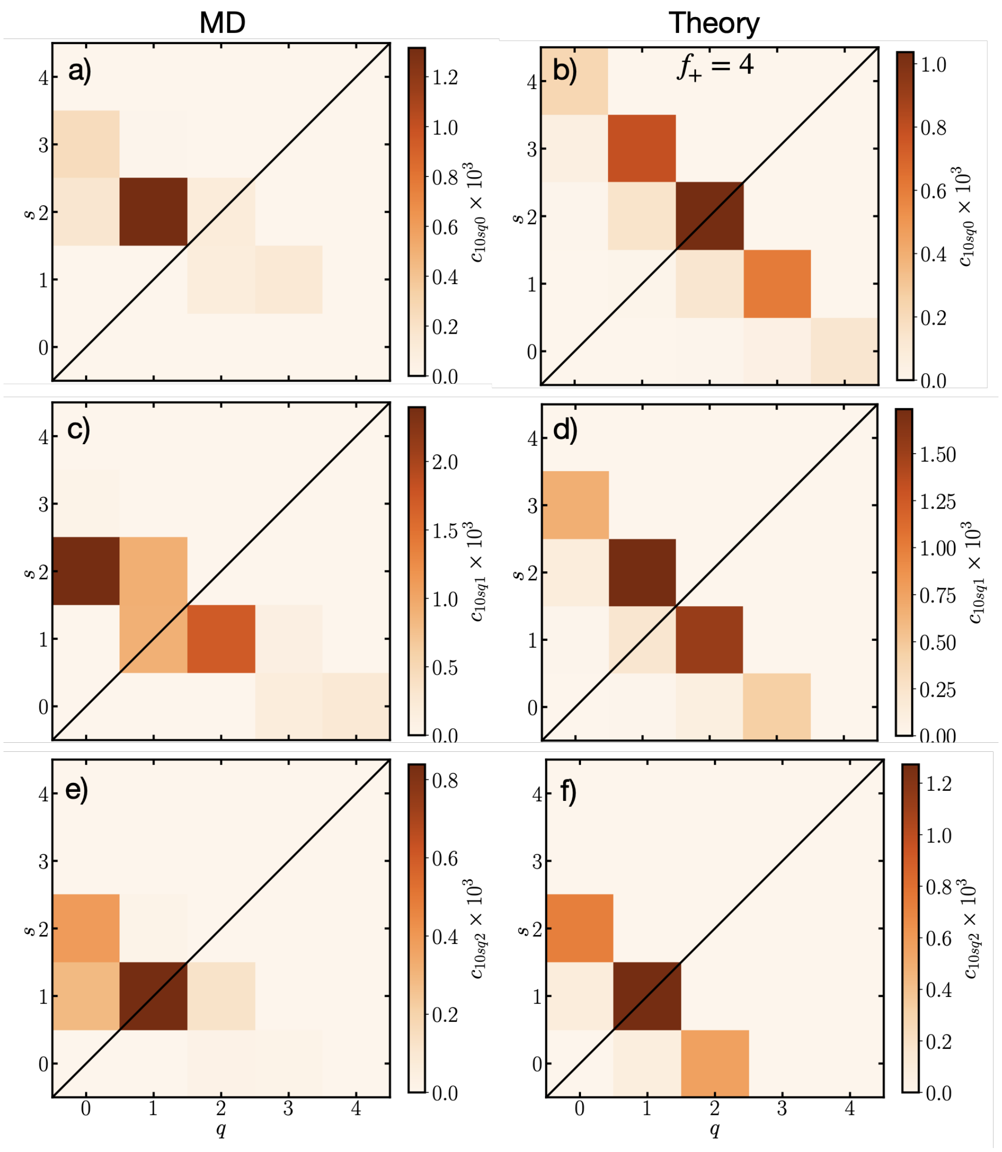


FIG. S25. Solvation distribution of Li, $c_{10sqp}$, in the diffuse layer from MD at $-0.6$ $e\text{nm}^{-2}$ [a),c),e)] and Theory [b),d),f)] as a function of the number of coordinating DME ($s$) and DOL ($q$) with, respectively, 0, 1 and 2 coordinating FEC solvents ($p$).

In Tab. S6 we report the parameters for the theory computed from MD. We find $\lambda_x/\lambda_y$ remains approximately constant, but $\lambda_x/\lambda_z$ reduces quite substantially, with the probabilities also changing quite a lot, indicating that again this system does not hold the assumptions of the simple theory in the main text.

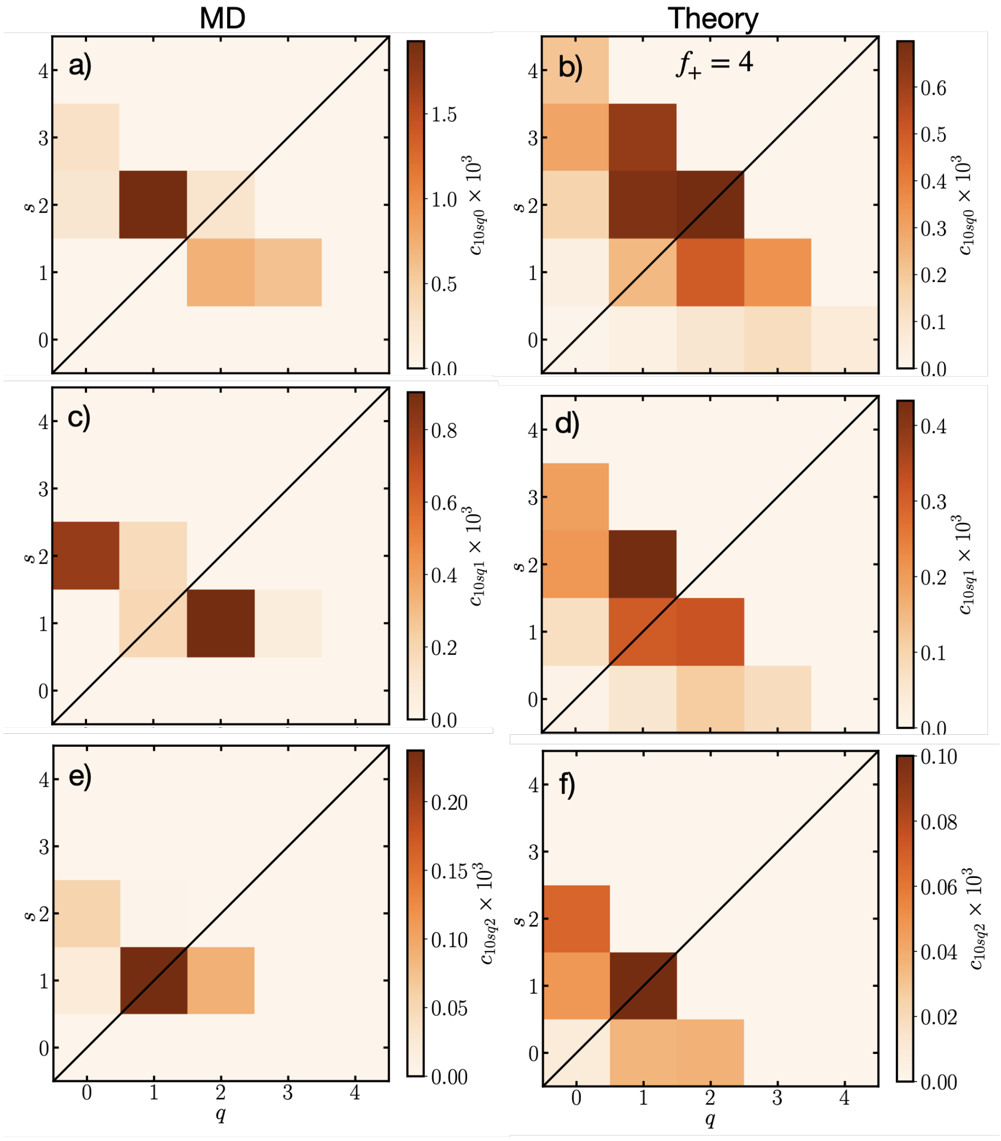


FIG. S26. Solvation distribution of Li, $c_{10sqp}$, in the diffuse layer from MD at $-0.8$ $e$nm$^{-2}$ [a),c),e)] and Theory [b),d),f)] as a function of the number of coordinating DME ($s$) and DOL ($q$) with, respectively, 0, 1 and 2 coordinating FEC solvents ($p$).

In Fig. S28 we show the solvent distributions in the Helmholtz layer at $-0.6$ $e$nm$^{-2}$. For this case, we use $f_+ = 2$ in the theory. The agreement for $c_{sq0}$ is reasonable. For $c_{sq1}$, there is significant $c_{021}$ in the MD simulation which the theory cannot capture as $f_+ = 2$. As the functionality is set to $f_+ = 2$, the the only solvation environment for $c_{sq2}$ is 2FEC, but in the MD simulations the most probable environment is found to be $c_{012}$. Overall, the theory match for $f_+ = 2$ with MD is mixed, but if $f_+ = 3$ was to be used it would also be mixed.

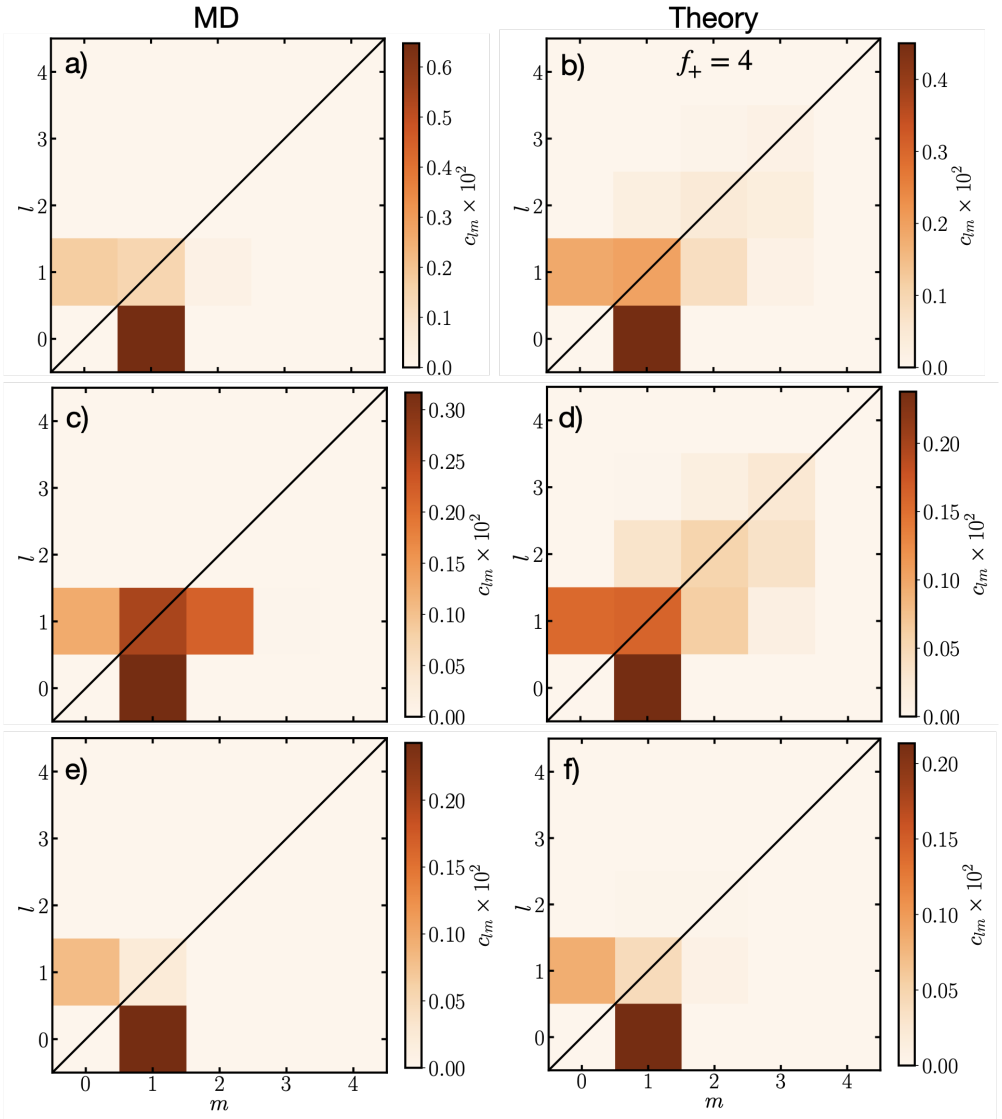


FIG. S27. Ionic aggregate distribution, $c_{lm}$, in the diffuse EDL from MD [a),c),e)] and theory [b),d),f)] as a function of the number cations ($l$) and anions ($m$) at, respectively, surface charges of +0.4, +0.6 and +0.8 $e$m$^{-2}$.

Finally, in Fig. S30 we show the solvent distributions in the Helmholtz layer at −0.8 enm$^{-2}$. In the theory we use $f_+ = 3$. In the MD simulations, for $p = 0$, practically all of the solvation environments are 3DOL, which the theory reasonably captures. For $p = 1$, the most likely environments in MD is 1DME and 2DOL. The theory, however, can only capture the significant concentration of 2DOL. Similarly, for $p = 2$, the theory predicts there to be more DOL environments than DME, but the MD prediction is reversed.

Overall, for DME-DOL-FEC, there appears to be a less strong reduction in the observed

| $\sigma$/ enm$^{-2}$ | $\lambda_x/\lambda_y$ | $\lambda_x/\lambda_z$ | $x_x/x_y$ | $x_x/x_z$ | $p_{+x}$ | $p_{+y}$ |
|---|---|---|---|---|---|---|
| -0.4 | 9.16 | 0.572 | 0.300 | 1.380 | 0.392 | 0.229 |
| -0.6 | 2.81 | 0.970 | 0.223 | 0.469 | 0.174 | 0.449 |
| -0.8 | 9.06 | 0.114 | 0.174 | 0.831 | 0.170 | 0.605 |

TABLE S6. Summary of association constant ratios and mole fraction ratios for the DME, DOL and FEC solvents for the Helmholtz layer at the indicated surface charges.

functionality in the Helmholtz layer, as it appears $f_+ = 3$ works well in the Helmholtz layer, and also in the bulk. However, as there is significant ion pairing in the bulk, using $f_+ = 4$ is perhaps more appropriate, but in the Helmholtz layer there is little/no anions, so using $f_+ = 3$ is more justified.

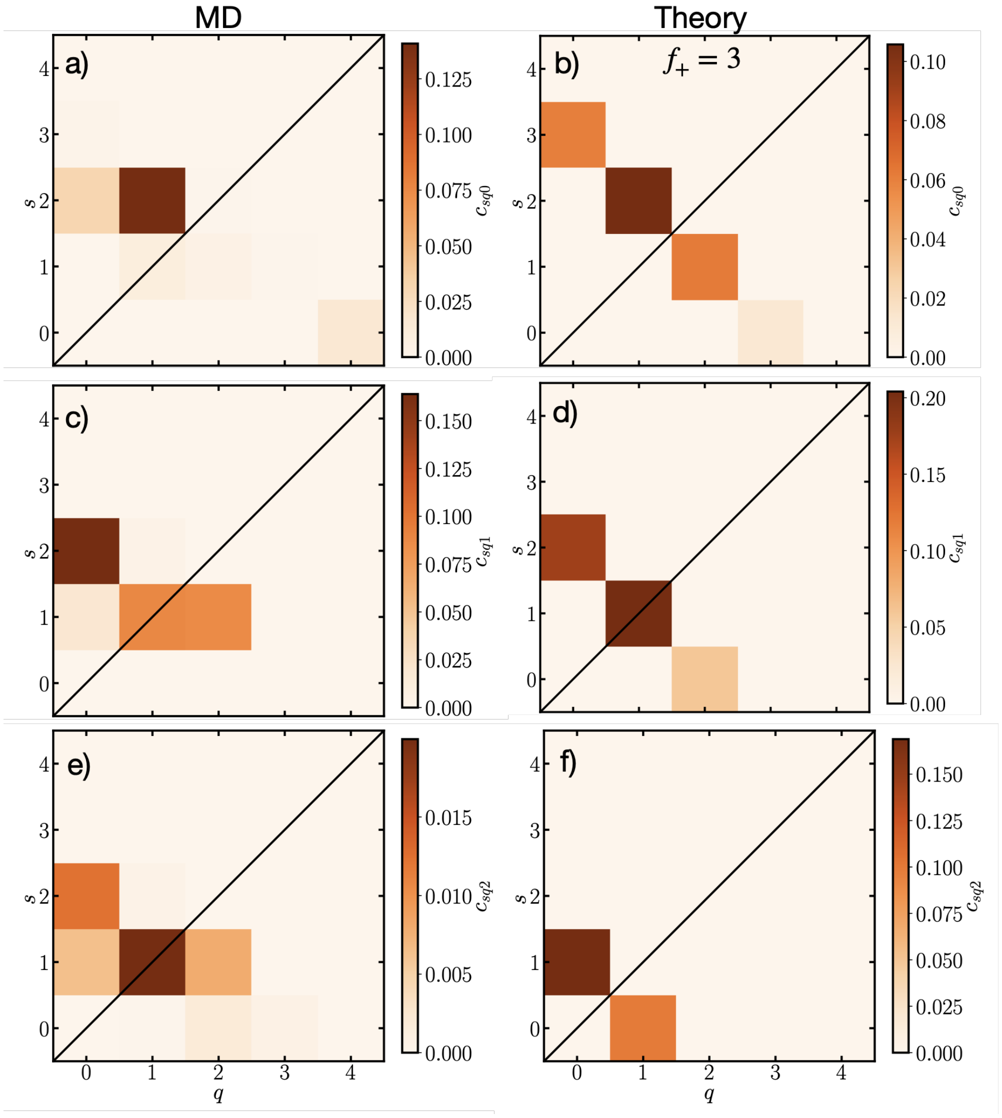


FIG. S28. Solvation distribution of Li, $c_{10sqp}$, in the Helmholtz layer from MD at $-0.4\ e\mathrm{nm}^{-2}$ [a),c),e)] and Theory [b),d),f)] as a function of the number of coordinating DME ($s$) and DOL ($q$) with, respectively, 0, 1 and 2 coordinating FEC solvents ($p$).

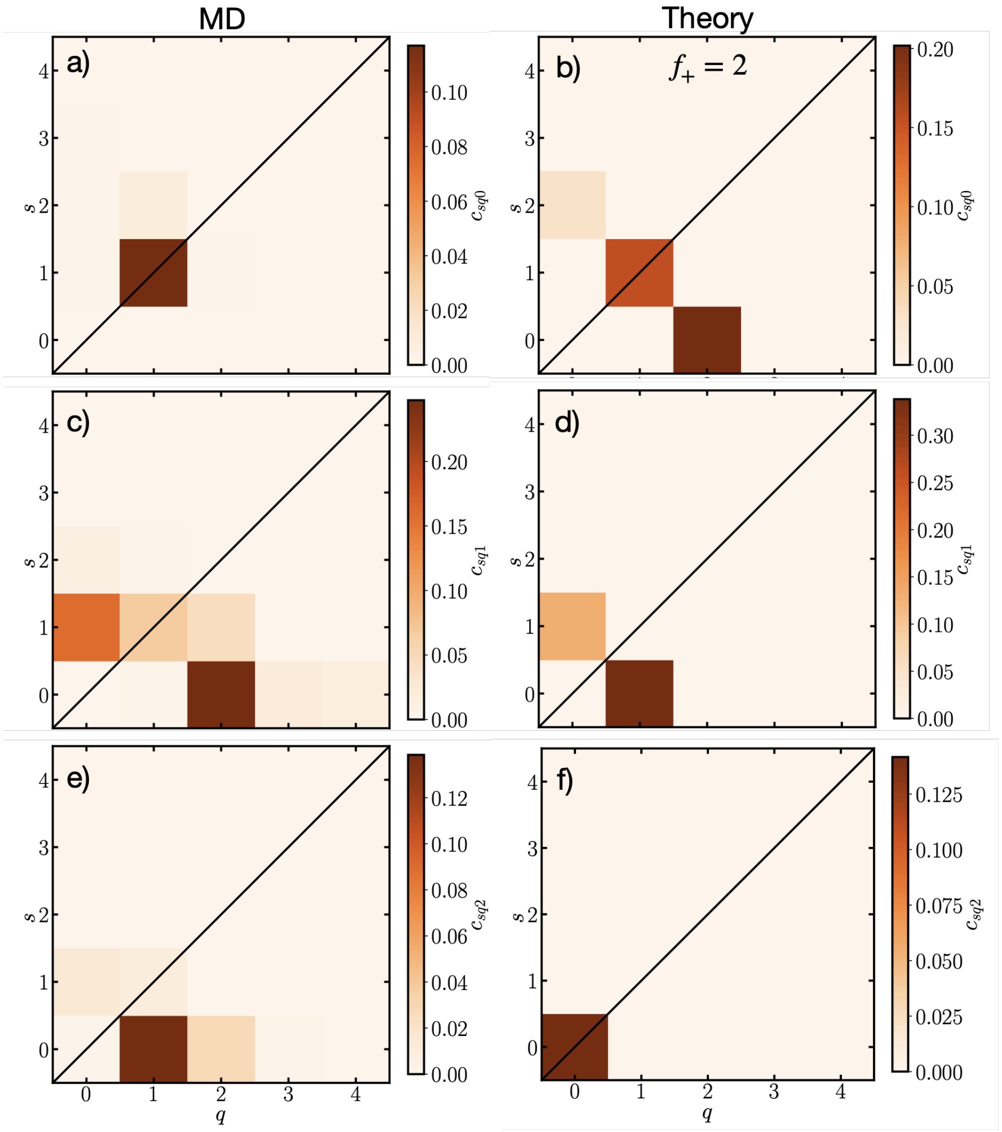


FIG. S29. Solvation distribution of Li, $c_{10sqp}$, in the Helmholtz layer from MD at $-0.6$ $e$nm$^{-2}$ [a),c),e)] and Theory [b),d),f)] as a function of the number of coordinating DME ($s$) and DOL ($q$) with, respectively, 0, 1 and 2 coordinating FEC solvents ($p$).

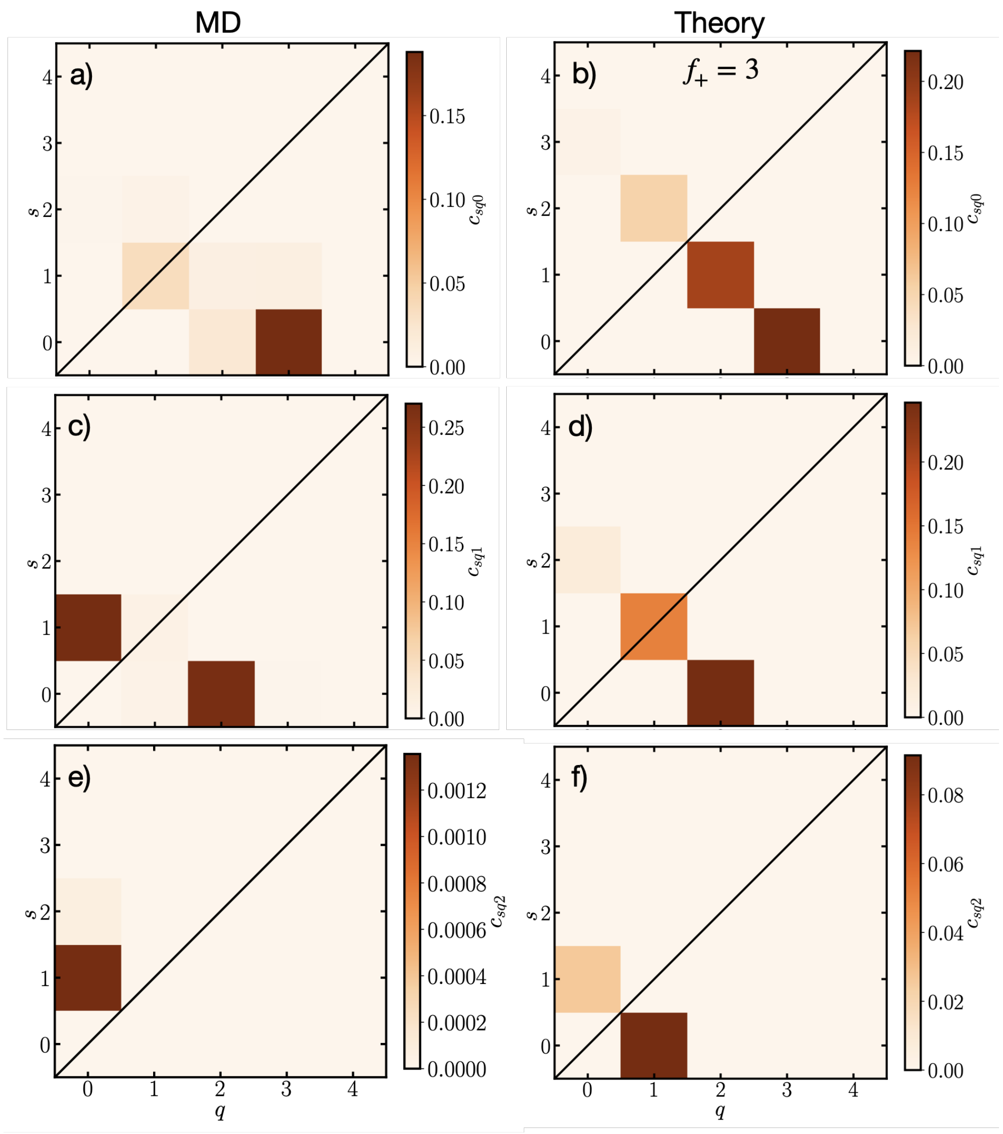


FIG. S30. Solvation distribution of Li, $c_{10sqp}$, in the Helmholtz layer from MD at $-0.8$ $e$nm$^{-2}$ [a),c),e)] and Theory [b),d),f)] as a function of the number of coordinating DME ($s$) and DOL ($q$) with, respectively, 0, 1 and 2 coordinating FEC solvents ($p$).

[1] D. M. Markiewitz, Z. A. H. Goodwin, M. McEldrew, J. P. de Souza, X. Zhang, R. M. Espinosa-Marzal, and M. Z. Bazant, Electric field induced associations in the double layer of salt-in-ionic-liquid electrolytes, Faraday Discuss. **253**, 365 (2024).

[2] D. M. Markiewitz, Z. A. H. Goodwin, Q. Zheng, M. McEldrew, R. M. Espinosa-Marzal, and M. Z. Bazant, Ionic associations and hydration in the electrical double layer of water-in-salt electrolytes, ACS Appl. Mater. Interfaces. **17**, 29515 (2025).

[3] Z. A. H. Goodwin, M. McEldrew, J. de Souza, M. Z. Bazant, and A. A. Kornyshev, Gelation, clustering and crowding in the electrical double layer of ionic liquids, J. Chem. Phys. **157**, 094106 (2022).
